# ANXA1-mediated mTOR/FABP4 Inhibition Drives Antifibrotic Macrophage Reprogramming in Lupus Nephritis

**DOI:** 10.7150/ijbs.118613

**Published:** 2026-02-04

**Authors:** Juan Tao, Qingyu Cheng, Pinjie Zhang, Guizhen Yu, Qi Chen, Manwen Yang, Qiqin Wu, Haopeng Fang, Haibo Wu, Xiaoyuan Song, Zhu Chen, Min Chen, Xiaoming Meng, Mingxing Lei, Tengchuan Jin

**Affiliations:** 1 Department of Rheumatology and Immunology, The First Affiliated Hospital of the USTC, Division of Life Sciences and Medicine, University of Science and Technology of China, Hefei 230001, China.; 2 Institute of Health and Medicine, Hefei Comprehensive National Science Center, Hefei 230071, China.; 3 Emergency Department, Peking University Shenzhen Hospital, Shenzhen 518000, China.; 4 Kidney Disease Center, the First Affiliated Hospital, College of Medicine, Zhejiang University; Key Laboratory of Kidney Disease Prevention and Control Technology, Zhejiang Province; Institute of Nephrology, Zhejiang University; Zhejiang Clinical Research Center of Kidney and Urinary System Disease, Hangzhou 310003, China.; 5 Department of Dermatology, The First Affiliated Hospital of the USTC, Division of Life Sciences and Medicine, University of Science and Technology of China, Hefei 230001, China.; 6 Division of Life Sciences and Medicine, University of Science and Technology of China, Hefei 230027, China.; 7 Department of Pathology, The First Affiliated Hospital of the USTC, Division of Life Sciences and Medicine, University of Science and Technology of China, Hefei 230001, China.; 8 MOE Key Laboratory for Membraneless Organelles and Cellular Dynamics, Hefei National Laboratory for Physical Sciences at the Microscale, CAS Key Laboratory of Brain Function and Disease, School of Life Sciences, Division of Life Sciences and Medicine, University of Science and Technology of China, Hefei 230026, China.; 9 Renal Division, Department of Medicine, Peking University First Hospital; Institute of Nephrology, Peking University; Key Laboratory of Renal Disease, Ministry of Health of China; Key Laboratory of Chronic Kidney Disease Prevention and Treatment (Peking University), Ministry of Education, Beijing 100034, China.; 10 Research Units of Diagnosis and Treatment of Immune-Mediated Kidney Diseases, Chinese Academy of Medical Sciences, Beijing 100730, China.; 11 Inflammation and Immune Mediated Diseases Laboratory of Anhui Province, the Key Laboratory of Anti-inflammatory of Immune Medicines, Ministry of Education, Anhui Institute of Innovative Drugs, School of Pharmacy, Anhui Medical University, Hefei 230032, China.; 12 Key Laboratory of Biorheological Science and Technology of Ministry of Education and 111 Project Laboratory of Biomechanics and Tissue Repair, College of Bioengineering, Chongqing University, Chongqing 400044, China.; 13 Laboratory of Structural Immunology, Key Laboratory of Immune Response and Immunotherapy, Division of Life Sciences and Medicine, University of Science and Technology of China, Hefei 230027, China.; 14 CAS Key Laboratory of Innate Immunity and Chronic Diseases School of Life Sciences and Medical Center, University of Science and Technology of China, Hefei 230027, China.; 15 Biomedical Sciences and Health Laboratory of Anhui Province, University of Science and Technology of China, Hefei 230027, China.; 16 Clinical Research Hospital of Chinese Academy of Sciences (Hefei), University of Science and Technology of China, Hefei 230001, China.

**Keywords:** lupus nephritis, macrophage, annexin A1, renal fibrosis, lipid metabolic reprogramming

## Abstract

Inflammation and fibrosis are central pathological processes in lupus nephritis (LN). Annexin A1 (ANXA1), a protein highly expressed in myeloid cells, is a key modulator of inflammation and fibrosis. In this study, we found that renal ANXA1 expression was elevated in LN patients and correlated positively with the severity of fibrosis. Single-cell RNA sequencing identified a distinct monocyte-derived *Anxa1^+^Spp1^+^* macrophage subset that expands during nephritis and displays a profibrotic transcriptional signature. Mechanistically, ANXA1 signals *via* the FPR2/ALX receptor to inhibit mTOR/FABP4 activity in macrophages, enhance fatty acid oxidation, and thereby drive a polarization shift toward an antifibrotic phenotype. Consequently, treatment with the ANXA1-mimetic peptide Ac2-26 attenuated macrophage-driven fibrosis, reduced renal lipid accumulation, and ameliorated kidney injury in lupus-prone mice. These findings underscore the critical role of ANXA1 and *Anxa1^+^Spp1^+^* macrophages in renal fibrosis progression, offering novel therapeutic targets for LN.

## 1. Introduction

Systemic lupus erythematosus (SLE) is a multisystem autoimmune disease characterized by pathogenic autoantibodies targeting nuclear antigens, immune complex deposition, and sustained inflammatory responses. Notably, 40-60% of SLE patients develop lupus nephritis (LN) 1, a serious renal complication of this systemic disorder. Despite substantial advances in diagnostic and therapeutic approaches for SLE/LN, the disease burden persists 2. Over a 10-year period, approximately 13% of LN patients develop end-stage renal disease (ESRD) 3, which is characterized by severe renal fibrosis and sclerosis. The standard medical regimens for LN, which typically entail the concomitant use of corticosteroids and immunosuppressants 2, 4, are associated with a substantial burden of adverse effects. Historically, clinical trials for SLE/LN have often yielded disappointing results 5. The urgency to develop innovative therapeutic strategies, especially those targeting novel immune checkpoints to facilitate precision medicine, is undeniable. Nevertheless, the cellular and molecular mechanisms underlying LN are still not fully understood 1, 6, 7.

Pathological fibrosis, characterized by excessive extracellular matrix (ECM) deposition, represents the terminal convergent mechanism underlying most chronic kidney disease (CKD) progression, with macrophages playing a crucial role in its pathogenesis 8-10. Our earlier studies established renal fibrosis as the paramount pathological feature correlating with poor renal survival in LN populations 11. Recent investigations have uncovered that metabolic reprogramming serves as a critical driver of renal fibrogenesis by disrupting cellular energetics and altering metabolite-mediated signaling cascades 8.

Annexin A1 (ANXA1), a 37-kDa calcium-dependent phospholipid-binding protein (previously termed lipocortin-1), is a glucocorticoid-inducible member of the annexin superfamily 12. This pleiotropic mediator regulates diverse physiological processes including inflammatory resolution, cell homeostasis (proliferation/apoptosis balance), and ECM remodeling 13, 14. It primarily expressed in myeloid cells, especially macrophages 13-15, exerts its effects through specific receptors such as FPR2/ALX, which are also predominantly found on macrophages 14, 16, 17. Interestingly, accumulating evidence indicates that ANXA1, originally characterized as an anti-inflammatory factor, has been increasingly recognized as a multifunctional regulator involved in fibrotic progression 14, 18 and metabolic homeostasis 19, 20. Despite its emerging role in LN, the specific contribution of ANXA1 to renal fibrosis in established disease remains ambiguous, highlighting a key area for translational research and therapeutic development.

In this study, we performed quantitative analyses of ANXA1 expression patterns in renal biopsy specimens from LN patients, and systematically correlated these patterns with clinical indices and histopathological features. Building on prior evidence of ANXA1 overexpression in myeloid compartments and the pivotal role of macrophages in CKD and LN pathogenesis 9, 21, 22, we utilized single-cell RNA sequencing (scRNA-seq) to characterize temporal heterogeneity in monocyte/macrophage populations at two distinct disease stages in MRL/*lpr* mice. This approach identified a pathogenic monocyte-derived macrophage subset (*Anxa1^+^Spp1^+^* macrophages) with distinctive profibrotic characteristics that exhibited disease-stage-specific expansion during LN progression, concomitant with upregulated *Anxa1* expression. Importantly, our findings demonstrate that ANXA1 may mitigate renal fibrosis in LN potentially by modulating the mTOR/FABP4 signaling pathway and improving fatty acid oxidation (FAO) in macrophages, thus driving the reprogramming of antifibrotic macrophages and curbing lipid accumulation. Furthermore, we evaluated the therapeutic efficacy of Ac2-26, a synthetic N-terminal ANXA1 peptide, in attenuating established nephritis in MRL/*lpr* lupus-prone mice.

## 2. Materials and methods

### 2.1 Experimental animals and materials

All experiments employed female MRL/*lpr* mice (SLAC Laboratory Animal Co., Shanghai) housed in specific pathogen-free conditions at the University of Science and Technology of China (USTC) Animal Center following institutional animal care protocols. For scRNA-seq, we examined pre-diseased mice aged 8 weeks without serum autoantibodies or proteinuria and nephritic mice ( > 18 weeks) with proteinuria levels ≥ 300 mg/dl persisting for more than two weeks (with four mice per group). For the treatment of MRL/*lpr* mice, 10-week-old MRL/*lpr* mice were subjected to Ac2-26 (2 mg/kg, in PBS) or vehicle (PBS) treatment (every 48 hours, intraperitoneally, with six mice per group). The Ac2-26 peptide was synthesized by MedChemExpress LLC (China), and the purity of Ac2-26 was 99.14%. After 10 weeks of administration, 24-hour urine was collected *via* a metabolic cage. Then, 20-week-old female MRL/*lpr* mice were fasted overnight and anesthetized. Whole blood (0.6-1.0 ml) was collected through the right atrium of the heart, and the kidneys were also collected. All animal experimental protocols were conducted following approval from the USTC Animal Ethics Committee (Ethical Approval No. USTCACUC26080122046). A comprehensive list of research antibodies and materials appears in Table S1.

### 2.2 Human subjects

Clinical samples were prospectively collected from the First Affiliated Hospital of USTC following institutional review board approval. Demographic and clinical characteristics of the LN cohort are systematically presented in Table S2, with comprehensive methodological details available in the Supplementary Methods section.

### 2.3 Single-cell RNA sequencing

scRNA-seq libraries were constructed using the Singleron GEXSCOPE platform following the manufacturer specifications. Briefly, cell suspensions (4×10^5^ cells/ml in PBS) were loaded onto microfluidic chips for single-cell capture using the Singleron Matrix system. Captured mRNA was reverse-transcribed from barcoded beads to generate first-strand cDNA, which was subsequently amplified by polymerase chain reaction (PCR). Following fragmentation and adapter ligation using the GEXSCOPE library preparation kit, final libraries were sequenced on an Illumina NovaSeq 6000 platform (150 bp paired-end).

### 2.4 Statistical analysis

Continuous variables are expressed as mean ± standard deviation for normally distributed data or median (interquartile range) for non-parametric distributions. Intergroup comparisons were performed using Student's *t*-test or Mann-Whitney *U* test, as appropriate based on data distribution characteristics. Statistical significance was defined as a two-tailed *P* value < 0.05. All analyses were conducted using SPSS Statistics (version 24.0, IBM Corp.) or R 23.

## 3. Results

### 3.1 Elevated ANXA1 expression correlates with renal fibrosis and impaired kidney function in patients with LN

**Figure 1:**
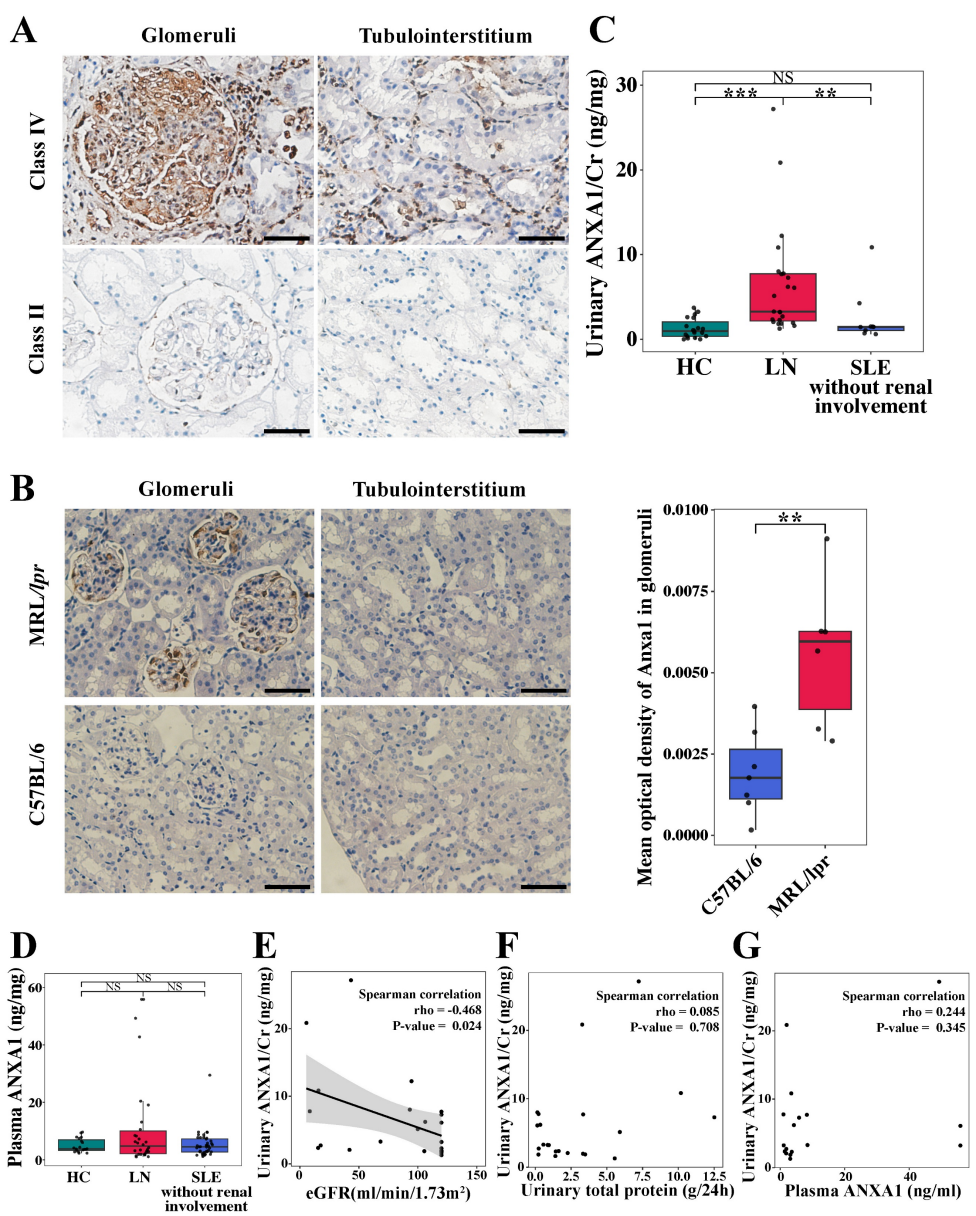
** Renal ANXA1 is significantly elevated in LN.** (A) Representative images of ANXA1 staining in the glomeruli and tubulointerstitium of human renal cortical tissue from LN patients with International Society of Nephrology/Renal Pathology Society class IV and class II disease. Bars = 50 μm. (B) Representative images of Anxa1 immunohistochemical staining of C57BL/6 (n = 7) and MRL/*lpr* (n = 6) mouse kidney sections and quantification of the results. Data analyses were performed by Student's *t*-test for two groups. Bars = 50 μm. (C) The level of urinary ANXA1/Cr in healthy controls (n = 19), LN patients (n = 23), and non-renal SLE patients (n = 9). Data analyses were performed by 1-way ANOVA followed by a Tukey test for 3 groups and expressed as mean ± SD. (D) The level of plasma ANXA1 in healthy controls (n = 19), LN patients (n = 30), and non-renal SLE patients (n = 40). Data analyses were performed by 1-way ANOVA followed by a Tukey test for 3 groups and expressed as mean ± SD. (E) Correlation analysis of the urinary ANXA1/Cr ratio and eGFR in LN patients (n = 23). (F) Correlation analysis of the levels of urinary ANXA1/Cr and total urinary protein in LN patients (n = 22). (G) Correlation analysis of the levels of urinary ANXA1/Cr and plasma ANXA1 in LN patients (n = 17). Correlations were all determined by Spearman analysis. *P < 0.05; **P < 0.01; ***P < 0.001. ANXA1: annexin A1; LN: lupus nephritis; Cr: creatinine; SLE: systemic lupus erythematosus; eGFR: estimated glomerular filtration rate.

Immunohistochemical staining was performed to quantify ANXA1 protein expression in renal biopsy specimens from LN patients. As shown in Figure 1A, ANXA1 levels were elevated in human LN tissues. Notably, in quantitative analyses across a cohort encompassing all LN classes, ANXA1 expression exhibited a progressive increase corresponding to the severity of renal injury in both glomerular and tubulointerstitial compartments (Figure 1A; Table 1; Table S3). The detailed histopathological classification is presented in Table S2. Furthermore, glomerular ANXA1 expression intensity demonstrated significant correlations with multiple clinicopathological parameters in LN patients (all *P* < 0.05; Table 1), including histological classification (nonproliferative vs proliferative), crescent formation (cellular/fibrocellular), NIH chronicity scores, glomerulosclerosis, and interstitial fibrosis. Comprehensive correlation analyses between tubulointerstitial ANXA1 expression patterns and clinicopathological parameters were conducted, with detailed results provided in Table S3. In complementary murine studies, renal Anxa1 expression remained undetectable in control C57BL/6 mice but was markedly upregulated in MRL/*lpr* glomeruli (Figure 1B). This injury-induced ANXA1 upregulation was further confirmed through quantitative image analysis (*P* < 0.01), establishing a consistent disease-associated expression pattern across human and murine models.

Quantitative analysis revealed distinct ANXA1 expression patterns in urine and plasma across study cohorts (Figure 1C and D). Urinary ANXA1/Cr ratios were significantly elevated in LN patients compared to both non-renal SLE patients (6.38 ± 6.43 vs. 2.60 ± 3.28 ng/mg, *P* < 0.01) and healthy controls (6.38 ± 6.43 vs. 1.28 ± 1.17 ng/mg, *P* < 0.001; Figure 1C). Notably, urinary ANXA1/Cr demonstrated a significant inverse correlation with estimated glomerular filtration rate (eGFR) (Spearman's rho = -0.468, *P* < 0.05), while showing no association with proteinuria (Spearman's rho = 0.085, *P >* 0.05) (Figure 1E and F). In contrast, plasma ANXA1 concentrations did not differ significantly among the three groups (Figure 1D). Furthermore, urinary ANXA1 excretion was not correlated with plasma ANXA1 levels (Spearman's rho = 0.244, *P* = 0.345) in LN patients (Figure 1G). These findings suggest that elevated ANXA1 is associated with renal fibrosis and dysfunction in LN.

### 3.2 Single-cell RNA sequencing revealed diverse monocyte/macrophage subpopulations in MRL/*lpr* mice

**Figure 2:**
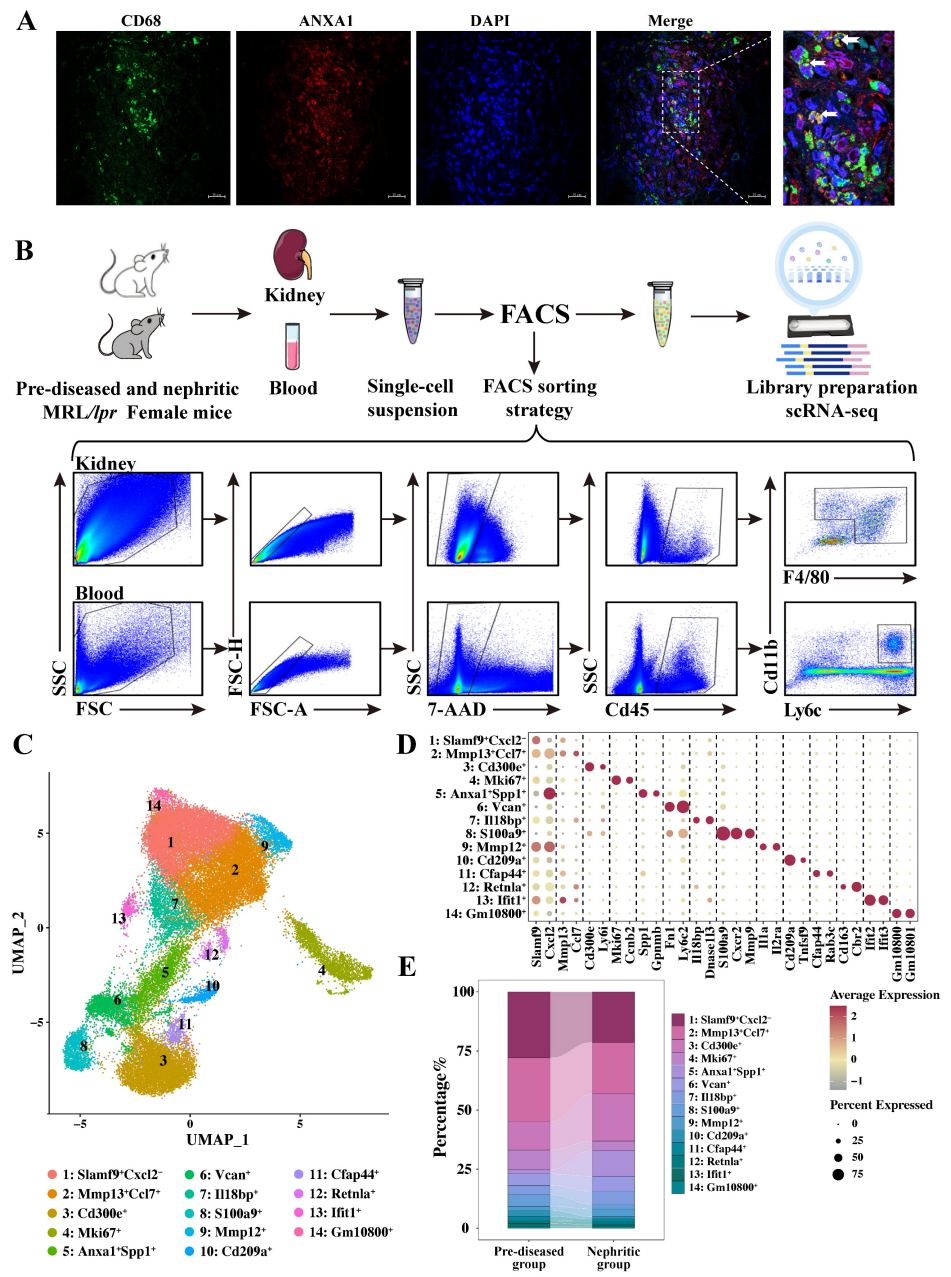
** Single-cell RNA sequencing revealed diverse monocyte/macrophage subpopulations in MRL/*lpr* mice.** (A) Representative confocal microscopy images showing the expression of ANXA1 in macrophages (CD68) in kidneys from patients with lupus nephritis. Bars = 20 μm. (B) Schematic of the single-cell RNA sequencing study design. Samples were isolated from the kidneys and blood of pre-diseased and nephritic MRL/*lpr* mice (n = 4 per group). Kidneys were digested into single-cell suspensions. Live cells were subjected to fluorescence-activated cell sorting and loaded for single-cell RNA sequencing. (C) Uniform manifold approximation and projection plot colored according to monocyte/macrophage clusters depicting monocyte/macrophage annotation. (D) Dot plot displaying the representative marker genes in each monocyte/macrophage subpopulation identified through unsupervised clustering. (E) Summary of the proportions of assigned cell types in the pre-diseased and nephritic groups.

Together with our earlier observations 22, the immunofluorescence staining results presented in Figure S1 further demonstrate a strong correlation between renal macrophage infiltration and disease progression in lupus nephritis. Furthermore, consistent with established literature 13-15, 24, analysis of a publicly available human LN dataset (GSE279823) by scRNA-seq demonstrated that ANXA1 is predominantly expressed in myeloid lineages, particular in macrophages, compared to other immune cell types (Figure S2). Immunofluorescence co-localization analysis revealed spatial association between ANXA1 and CD68⁺ macrophages in renal biopsy specimens from LN patients (Figure 2A). These findings suggest that macrophage-derived ANXA1 may represent a key modulator in the pathogenesis of LN, potentially serving as a therapeutic target for modulating macrophage-mediated inflammatory and fibrotic responses.

We conducted scRNA-seq analysis on fluorescence-activated cell sorting (FACS)-purified live cells obtained from kidney and blood samples of female MRL/*lpr* mice at two distinct disease timepoints: pre-diseased and established nephritis (Figure 2B). Using the Singleron microfluidic chip platform, we characterized 116,950 sorted cells after applying quality control filters, which included 55,676 pre-diseased and 61,274 nephritic mouse single cells. Integrated single-cell transcriptomes were visualized using uniform manifold approximation and projection (UMAP) following batch correction, revealing 15 transcriptionally distinct clusters through unsupervised clustering (Figure S3A). Cell cluster identities were annotated based on statistically significant expression of established lineage-specific markers (Figure S3B). Subsequent analysis of monocyte/macrophage markers (*Adgre1*, *Cx3cr1*, *Csf1r*, *Cd14*, *Fcgr3*, and *C1qa*; Figure S3C) identified 37,143 cells as monocytes/macrophages.

To further characterize their heterogeneity and phenotype, these monocytes/macrophages were subdivided into 14 transcriptionally distinct subpopulations based on different markers (Figure 2C, D, and Table S4). Nonlinear dimensionality reduction via UMAP revealed distinct transcriptional landscapes of monocyte/macrophage subpopulations at each stage (Figure S3D). A remarkably heterogeneous distribution of cells at each stage was primarily identified within individual monocyte/macrophage subsets (Figure 2E, Table S5). In response to nephritis, a decrease in the cellular proportion was observed in Clusters 1, 2, 4, 8, 10, 11, 13, and 14, whereas an increase was observed in Clusters 3, 6, 7, 9, and 12, with a particularly sharp six-fold increase in Cluster 5. These shifts provide valuable insights into the dynamic changes in monocyte/macrophage populations during the progression of LN.

### 3.3 The origin of monocyte/macrophage subpopulations during LN disease progression

**Figure 3:**
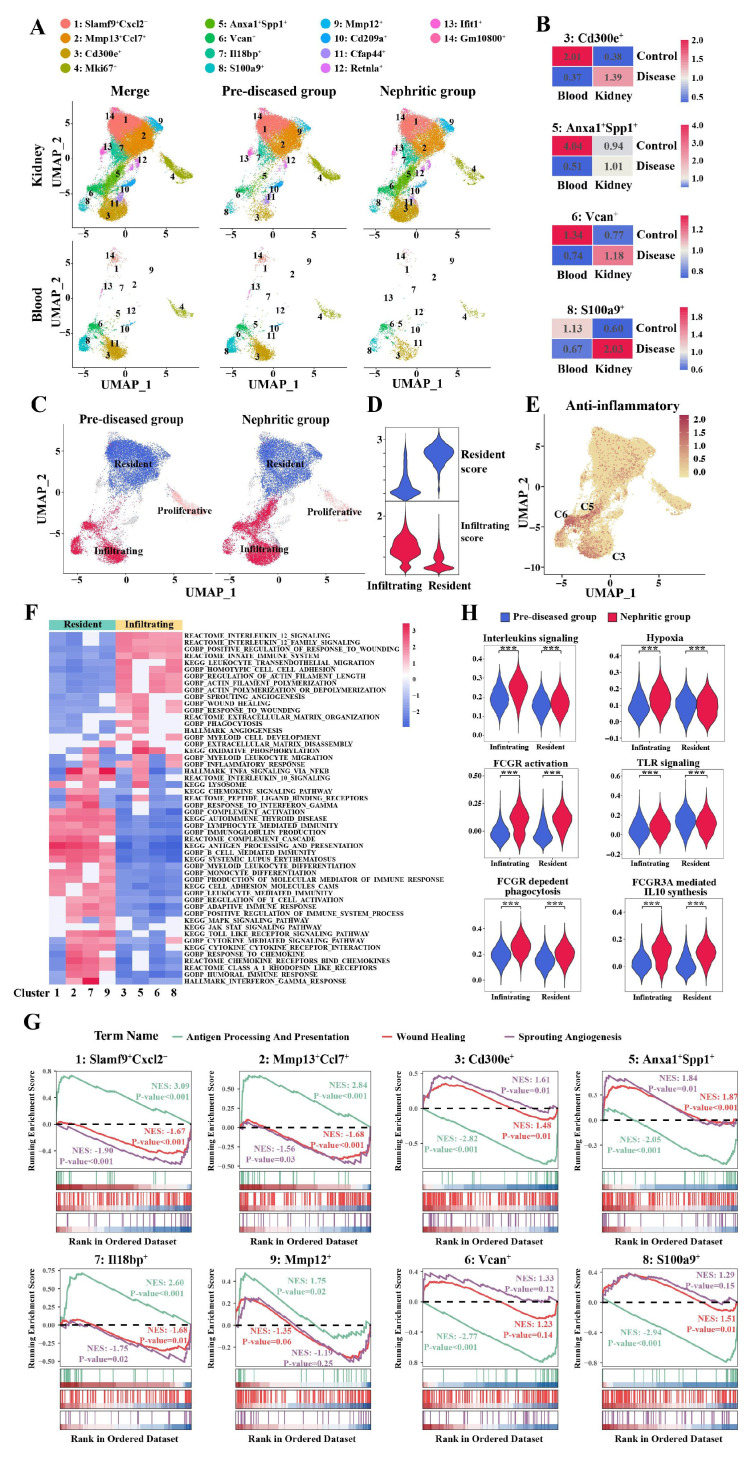
** The origin and functional heterogeneity of monocyte/macrophage subpopulations during lupus nephritis disease progression.** (A) Uniform manifold approximation and projection plots demonstrating the distribution of monocyte/macrophage clusters across two distinct stages and organs. (B) The tissue prevalence of each monocyte-derived infiltrating macrophage cluster was estimated by the Ro/e score. (C) Uniform manifold approximation and projection plots demonstrating the resident and infiltrating monocytes/macrophages. (D) Violin plots demonstrating the resident and infiltrating scores of the monocytes/macrophages. (E) Uniform manifold approximation and projection plots demonstrating the anti-inflammatory scores of the monocytes/macrophages. (F) Heatmap illustrating the normalized enrichment scores in gene set enrichment analysis for each predefined gene set, with the color gradient representing the normalized enrichment scores from negative (blue) to positive (red) values. (G) Illustration of the gene set enrichment analysis results for three specific biological processes, including antigen processing and presentation (green), wound healing (red), and sprouting angiogenesis (purple). (H) Violin plots illustrating the expression of six curated gene signatures in infiltrating and resident macrophages across two distinct stages. ***P < 0.001.

Systematic evaluation of established infiltrating versus resident monocyte/macrophage markers, combined with tissue enrichment analysis, revealed distinct spatial partitioning of monocyte/macrophage subpopulations across defined clusters (Figure 3A). Clusters 1, 2, 7, and 9 were enriched predominantly in kidney samples but were nearly absent in blood samples. These clusters were the primary monocyte/macrophage clusters present during homeostasis (pre-diseased) and persisted in the nephritic kidney. Notably, Clusters 3, 5, 6, and 8 demonstrated conserved transcriptional profiles across compartments, maintaining topographical alignment between renal and circulatory UMAP embeddings. Taking these observations into account, along with the expression levels of infiltrating-resident markers (Figure S4A, B) and the Ro/e values (Figure 3B), Clusters 3, 5, 6, and 8 were defined as monocyte-derived infiltrating macrophages, whereas Clusters 1, 2, 7, and 9 were defined as kidney-resident macrophages. The Ro/e values more clearly indicated the recruitment of monocytes from the peripheral blood to the kidney in response to injury. This classification result was further supported by the infiltration and resident scores based on infiltrating-resident markers (Figure 3C, D). The relative cell abundance of each assigned typical infiltrating and resident macrophage cluster in the kidney in the pre-diseased and nephritic groups is shown in Table S6.

Among the infiltrating macrophages, Cluster 6 was characterized by the typical monocyte markers *Ly6c2* and* Ccr2* and the phagocytic partner *Msr1* (Figure S5A, B). Cluster 3 demonstrated reduced *Ly6c2* expression and specific expression of *Cd36*, a marker of M2 macrophages, as well as high expression of *Nr4a1* and *Itgal* (Figure S5A-D). Cluster 5 was also characterized by decreased expression of *Ly6c2* (Figure S5A, B) and high expression of both *Anxa1* and *Spp1*, which was subsequently described. Intriguingly, Clusters 3, 5, and 6 presented distinct anti-inflammatory signatures (Figure 3E).

In addition to the monocyte marker genes *Ly6c2* and* Ccr2,* Cluster 8 uniquely displayed neutrophil-related genes *S100a8* and *S100a9,* but lacked *Ly6g* expression (Figure S5A, B, E, F). This signature suggests a granulocyte-monocyte progenitor (GMP)-derived neutrophil-like monocyte population 25, 26. Consistent with this finding, Cluster 8 also highly expressed the inflammation-related genes *Mmp9, Mmp8,* and* Il1r2*, which strongly reflected the proinflammatory macrophage phenotype (Figure S5E, F). This detailed characterization of monocyte/macrophage subpopulations provides a deeper understanding of their distinct functional contributions to LN pathogenesis.

### 3.4 The functional heterogeneity of monocyte/macrophage subpopulations

To further delineate functional heterogeneity among individual macrophage/monocyte clusters, we performed gene set enrichment analysis (GSEA). Among the kidney-resident macrophages, GSEA revealed significant enrichment of biological functions related to antigen processing/presentation, complement, chemokine signaling, proinflammatory signaling pathways, and Toll-like receptor (TLR) signaling (Figure 3F, G).

Among the infiltrating macrophages, Clusters 3, 5, 6, and 8 were primarily involved in IL-12 signaling and wound healing, whereas the activation of actin filament polymerization and filament length regulation was observed in these infiltrating subsets, with the exception of Cluster 5 (Figure 3F, G). In particular, Cluster 3 (*Cd300e^+^*) was also involved in phagocytosis and myeloid cell development, whereas Cluster 5 (*Anxa1^+^Spp1^+^*) and Cluster 6 (*Vcan^+^*) were involved in oxidative phosphorylation (Figure 3F), supporting their anti-inflammatory properties. In addition to being involved in wound healing and angiogenesis, Cluster 5 (*Anxa1^+^Spp1^+^*) also exhibited significant enrichment in biological functions related to lysosomes, IL-10 signaling, myeloid leukocyte migration, and ECM organization (Figure 3F). Cluster 8 (*S100a9^+^*) was enriched essentially in the cytokine-mediated signaling pathway and leukocyte transendothelial migration (Figure 3F).

During the progression of LN, infiltrating macrophages exhibited enhanced activation of key inflammatory pathways, including interleukin signaling, hypoxia response, Fcγ receptor-mediated signaling, and TLR pathways (Clusters 3, 5, 6, and 8) (Figure 3H). These findings demonstrate that both kidney-resident and infiltrating macrophages synergistically promote LN progression. Notably, infiltrating macrophages appear to function as primary pathogenic mediators, orchestrating critical molecular pathways that drive disease initiation and progression.

### 3.5 *Anxa1^+^Spp1^+^* macrophages that accumulate in the nephritic kidney have profibrotic properties

**Figure 4:**
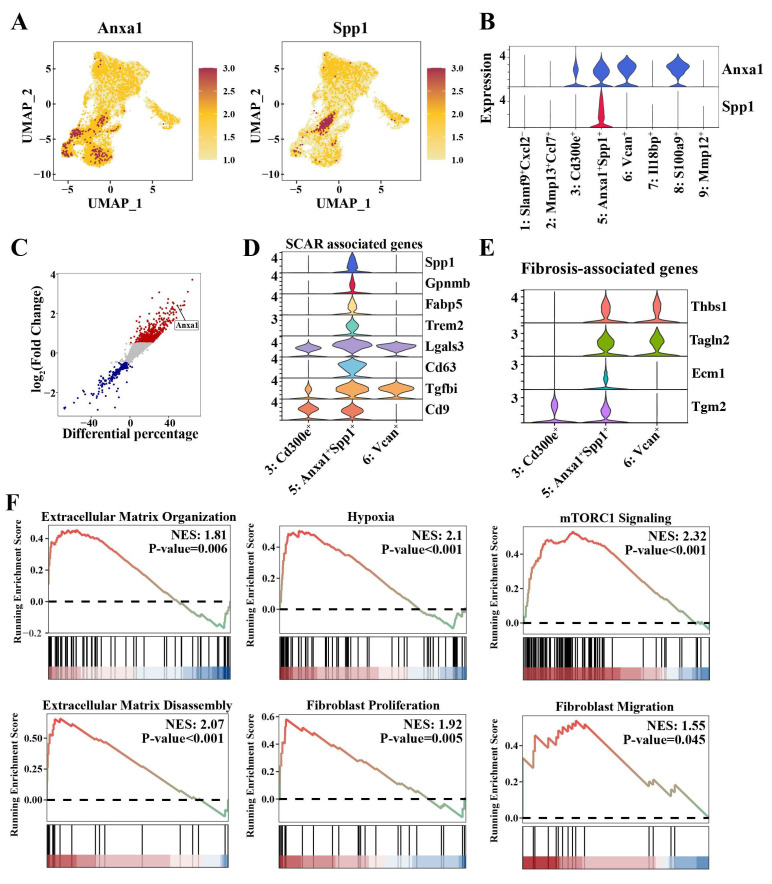
***Anxa1^+^Spp1^+^* macrophages that accumulate in nephritis kidneys have profibrotic properties.** (A) Uniform manifold approximation and projection plots demonstrating the key markers (*Anxa1* and *Spp1*) in Cluster 5 (*Anxa1^+^Spp1^+^*). (B) Expression of *Anxa1* and *Spp1* in distinct macrophage subsets. (C) Illustration of genes differentially expressed between resident and infiltrating macrophages. Each data point represents a gene, with the x-axis representing the differential percentage of cells expressing the corresponding gene. The y-axis represents the log2-fold change in gene expression. Genes significantly differentially expressed are highlighted, with upregulated genes in red and downregulated genes in blue. (D) Violin plots illustrating the expression of scar-associated genes across three monocyte-derived infiltrating macrophage subsets. (E) Violin plots illustrating the expression of fibrosis-associated genes across three monocyte-derived infiltrating macrophage subsets. (F) Gene set enrichment analysis results for six specific biological processes, including extracellular matrix (ECM) organization, hypoxia, mammalian target of rapamycin complex 1 signaling, ECM disassembly, fibroblast proliferation, and fibroblast migration, in Cluster 5 (*Anxa1^+^Spp1^+^*).

Intriguingly, our comprehensive monocyte/macrophage atlas revealed a distinct Cluster 5 (*Anxa1^+^Spp1^+^*) that was markedly expanded in the nephritic kidney but scarcely detectable in the pre-diseased kidney (Figure 3A and Table S6). This cluster was defined by the co-expression of *Anxa1* and *Spp1* (Figure 4A, B). Notably, *Anxa1* was among the most prominently upregulated genes in infiltrating macrophages relative to resident macrophages (Figure 4C). The “*Anxa1^+^Spp1^+^*” nomenclature thus highlights this specific co-expression pattern as a defining feature of the subset. As mentioned above, Cluster 5 (*Anxa1^+^Spp1^+^*) cells co-expressed canonical monocyte markers, including *Ly6c2*, *Ccr2*, and *Msr1* (Figure S5A-B), suggesting their origin from monocyte-derived infiltrating macrophages that undergo phenotypic adaptation following renal injury. Notably, Cluster 5 (*Anxa1^+^Spp1^+^*) represented the most responsive macrophage subset during nephritis progression, demonstrating a six-fold increase in renal infiltration in the nephritic group compared to the pre-diseased cohort (Table S6).

Cluster 5 (*Anxa1^+^Spp1^+^*) exhibited anti-inflammatory signatures similar to those of Cluster 3 (*Cd300e^+^*) and Cluster 6 (*Vcan^+^*) (Figure 3E). Nonetheless, this cluster was clearly distinguished from these two anti-inflammatory clusters through its elevated expression of a suite of scar-associated genes, including *Spp1*, *Gpnmb*, *Fabp5*, *Trem2*, *Lgals3*,* Cd63, Tgfbi,* and* Cd9* (Figure 4D). In addition, Cluster 5 (*Anxa1^+^Spp1^+^*) exhibited a pro-fibrotic transcriptomic signature, encompassing TGF-β-regulated genes (*Thbs1*), canonical myofibroblast markers (*Tagln2*), and genes encoding ECM components (*Ecm1*) and ECM crosslinkers (*Tgm2*) (Figure 4E).

Furthermore, hypoxia signaling, which plays a major role in triggering and perpetuating profibrotic mechanisms, was highly activated in this cluster (Figure 4F). More importantly, ECM organization and disassembly and fibroblast proliferation and migration were also activated in Cluster 5 (*Anxa1^+^Spp1^+^*) (Figure 3F, 4F). Notably, the mammalian target of rapamycin complex 1 (mTORC1) signaling was also activated in Cluster 5 (*Anxa1^+^Spp1^+^*) (Figure 4F). To investigate the direct role of *Spp1*, we established a stable *Spp1*-overexpressing RAW264.7 macrophage line via lentiviral transduction. *Spp1* overexpression promoted a profibrotic signature, characterized by increased mRNA expression of *Lgals3* and* Tgf-β* and elevated protein levels of collagen I, fibronectin, and α-SMA (Figure S6). Collectively, these findings indicate that Cluster 5 (*Anxa1^+^Spp1^+^*) may possess profibrotic functions and potentially initiate fibrosis.

### 3.6 Effects of Anxa1 on the dynamic functional plasticity of monocyte-derived macrophages

**Figure 5:**
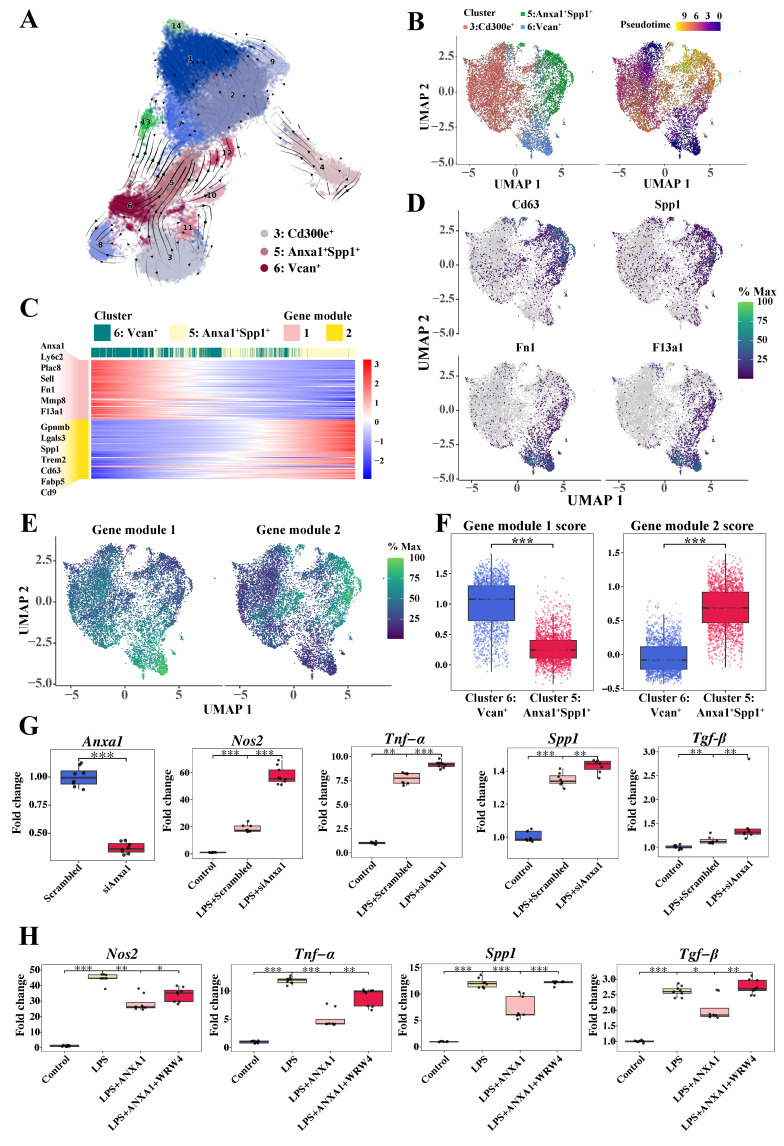
** Dynamic functional plasticity of monocyte-derived macrophages and the effects of Anxa1 on macrophage polarization.** (A) RNA velocity analysis *via* scVelo, illustrating the pseudotime trajectory of cells on the basis of their gene expression profiles. The arrows indicate the direction and magnitude of the RNA velocity, suggesting the likely future state of each cell along the trajectory. (B) Monocle 3 trajectory analysis of three monocyte-derived infiltrating macrophage subsets, including Cluster 3 (*CD300e^+^*), Cluster 5 (*Anxa1^+^Spp1^+^*), and Cluster 6 (*Vcan^+^*). The uniform manifold approximation and projection plot on the left displays three distinct subsets, and the cells in the right plot are elegantly color-coded to reflect their corresponding pseudotime. (C) Heatmap illustrating the patterns of gene expression within the two modules over pseudotime. (D) Uniform manifold approximation and projection plots illustrating the expression profiles of *Cd63*, *Spp1*, *Fn1*, and *F13a1*. (E) Uniform manifold approximation and projection plots illustrating the expression profiles of two gene modules. (F) Box plots illustrating the expression profiles of two gene modules in Cluster 5 (*Anxa1^+^Spp1^+^*) and Cluster 6 (*Vcan^+^*). (G) Quantitative real-time polymerase chain reaction (RT-qPCR) analysis of *Anxa1*, *Nos2*, *Tnf-α*, *Spp1*, and *Tgf-β* mRNA levels in RAW264.7 macrophages transfected with *Anxa1* small interfering RNA under LPS stimulation. n = 9 per group. (H) RT-qPCR analysis of *Nos2*, *Tnf-α*, *Spp1*, and *Tgf-β* mRNA levels in RAW264.7 macrophages treated with 10 nM human recombinant ANXA1 for 24 h with or without WRW4 under LPS stimulation. n = 9 per group. Data analyses were performed by Student's *t*-test for two groups and two-way ANOVA followed by a Tukey test for multiple groups. *P < 0.05; **P < 0.01; ***P < 0.001.

Considering the microenvironment-dependent plasticity of macrophages, we performed RNA velocity analysis and pseudotime trajectory inference using scVelo 27 and Monocle 3 28 (Figure 5A, B). RNA velocities, as vectors in gene expression space, signify the direction and speed of cellular movement, providing an approximation of future transcriptional states. As shown in Figure 5A, the velocities were projected onto the UMAP embedding, distinctly illustrating the velocity vector directions from Cluster 3 (*Cd300e^+^*) and Cluster 6 (*Vcan^+^*) toward Cluster 5 (*Anxa1^+^Spp1^+^*). Accordingly, these three monocyte-derived infiltrating macrophage clusters were selected for subsequent analysis. We used Monocle 3 to construct a specific trajectory from Cluster 6 (*Vcan^+^*) and Cluster 3 (*Cd300e^+^*) toward Cluster 5 (*Anxa1^+^Spp1^+^*) (Figure 5B).

Given that Cluster 6 exhibited characteristic expression of monocyte markers *Ly6c2* and *Ccr2*, we performed a comprehensive trajectory analysis to delineate the differentiation trajectory from Cluster 6 (*Vcan^+^*) to Cluster 5 (*Anxa1^+^Spp1^+^*). Gene dynamics changes alongside pseudotime, which were divided into two modules (Figure 5C), are illustrated. The M2 macrophage-related anti-inflammatory markers predominant in module 1, including *Fn1* and *F13a1,* were highly expressed at the beginning of the trajectory, where Cluster 6 (*Vcan*^+^) was located, but gradually decreased alongside the pseudotime. Conversely, fibrosis-related markers predominant in module 2, including *Cd63*, *Spp1*, and *Gpnmb*, were initially expressed at low levels but increased to relatively high levels toward the end of the trajectory, where Cluster 5 (*Anxa1*^+^*Spp1*^+^) was preferentially located. Dynamic gene expression changes associated Cluster 6 (*Vcan*^+^) with an anti-inflammatory role and Cluster 5 (*Anxa1*^+^*Spp1*^+^) with a profibrotic role, pointing to phenotypic and functional transitions of these macrophages following renal infiltration.

To elucidate the above dynamic changes more clearly, we performed pseudotime trajectory analysis tracking representative marker expression dynamics (Figure 5D). This analysis revealed a coordinated cellular transition from the M2 anti-inflammatory phenotype to the profibrotic phenotype. Consistently, as shown in Figure 5E-F, gene module 1 scores progressively decreased, while module 2 scores increased within Cluster 5 (*Anxa1^+^Spp1^+^*), indicating a phenotypic switch from anti-inflammatory to profibrotic gene expression programs.

Collectively, these findings revealed that anti-inflammatory macrophages (Cluster 6 (*Vcan^+^*)) are likely to differentiate into newly appearing profibrotic macrophages (Cluster 5 (*Anxa1^+^Spp1^+^*)) under ongoing unresolved insult in the context of lupus, corroborating our prior clinical observations of M2 macrophage predominance in the human LN 22.

Furthermore, to explore the role of Anxa1 in the phenotypic conversion of macrophages, RAW264.7 macrophages were used to assess Anxa1-mediated polarization dynamics. Knocking down *Anxa1* resulted in the upregulation of proinflammatory (*Nos2* and *Tnf-α*) and profibrotic factors (*Spp1* and *Tgf-β*) in response to LPS stimulation (Figure 5G). Treatment with human recombinant Annexin A1 protein (hrANXA1) significantly downregulated expression of proinflammatory (*Nos2* and *Tnf-α*) and profibrotic genes (*Spp1* and *Tgf-β*) (Figure 5H). Both previous studies 14, 16, 17 and our data demonstrate an interaction between ANXA1 and FPR2/ALX in macrophages (Figure S7). Pharmacological inhibition of FPR2/ALX using WRW4 (a selective FPR2/ALX antagonist) abrogated hrANXA1-mediated suppression of both inflammatory and fibrotic markers, establishing FPR2/ALX as the essential receptor for ANXA1's bioactivity (Figure 5H). The findings were further validated using bone marrow-derived macrophages (BMDMs), with complementary ELISA quantifying the secretion of Tnf-α and Tgf-β (Figure S8). Collectively, these data demonstrate that ANXA1 orchestrates macrophage polarization through FPR2/ALX-mediated signaling, while concomitantly suppressing the expression of fibrogenic markers, thereby promoting an anti-fibrotic transcriptional program.

### 3.7 Anxa1 regulates macrophage polarization through lipid metabolic reprogramming

**Figure 6:**
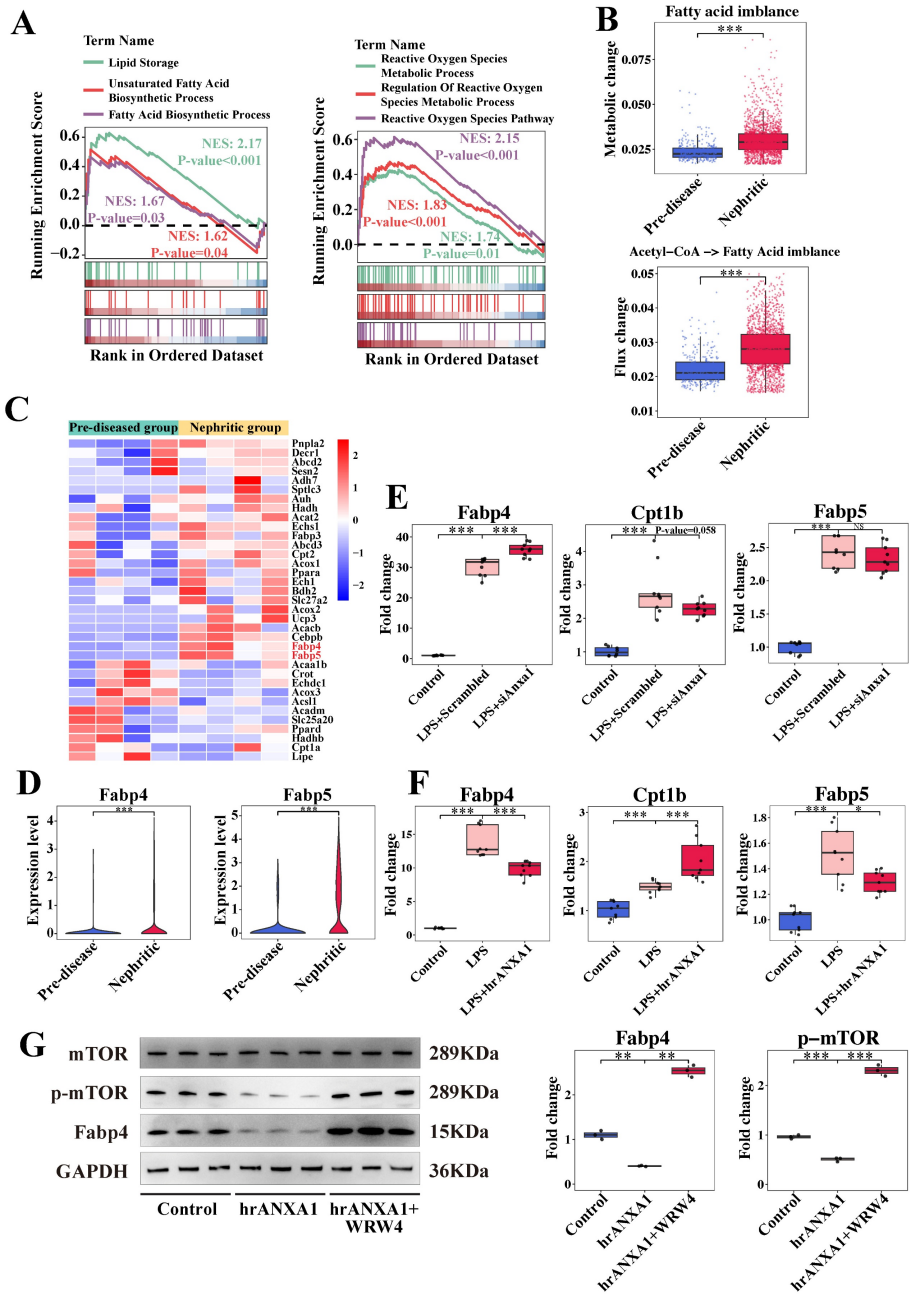
** ANXA1 might regulate macrophage polarization through lipid metabolism reprogramming.** (A) Illustration of the gene set enrichment analysis results in Cluster 5 (*Anxa1^+^Spp1^+^*) for six specific biological processes, including lipid storage, unsaturated fatty acid biosynthetic processes, fatty acid biosynthetic processes, reactive oxygen species metabolic processes, regulation of reactive oxygen species metabolic processes, and the reactive oxygen species pathway. (B) Predicted metabolic stress (upper) and flux changes (lower) in the pre-diseased group versus the nephritic group. (C) Heatmap illustrating the expression patterns of several lipid metabolisms reprogramming-associated genes between the pre-diseased and nephritic groups. (D) Violin plots illustrating the differential expression of *Fabp4* and *Fabp5* between the pre-diseased and nephritic groups. (E) Quantitative real-time polymerase chain reaction (RT-qPCR) analysis of *Cpt1b*, *Fabp4*, and *Fabp5* mRNA levels in RAW264.7 macrophages transfected with *Anxa1* small interfering RNA under LPS stimulation. n = 9 per group. (F) RT-qPCR analysis of *Cpt1b*, *Fabp4*, and *Fabp5* mRNA levels in RAW264.7 macrophages treated with 10 nM human recombinant ANXA1 (hrANXA1) for 24 h under LPS stimulation. n = 9 per group. (G) Representative western blot bands and densitometric quantification of the expression of Fabp4 and the phosphorylation of the mammalian target of rapamycin (mTOR) in RAW 264.7 macrophages treated with 10 nM hrANXA1 for 24 h with or without WRW4. n = 3 per group. Data analyses were performed by Student's *t*-test for two groups and two-way ANOVA followed by a Tukey test for multiple groups. *P < 0.05; **P < 0.01; ***P < 0.001.

Based on established roles of lipid metabolism in renal fibrogenesis 29, 30 and the known metabolic reprogramming of macrophages within the LN microenvironment 21, we focused on lipid- and fatty acid-related pathways in Cluster 5 (*Anxa1^+^Spp1^+^*) macrophages. Our observations revealed the activation of lipid storage, fatty acid biosynthesis, and reactive oxygen species metabolic processes within this cluster (Figure 6A). By analyzing the metabolic stress in this cluster, we noted a fatty acid metabolic imbalance, indicated by the accumulation of fatty acids in the nephritic group compared to pre-diseased controls (Figure 6B). In addition, increased metabolic flux from acetyl-CoA to fatty acids in the nephritic group was consistent with this accumulation (Figure 6B).

Transcriptomic profiling identified significant upregulation of fatty acid-binding proteins, such as *Fabp4* and *Fabp5*, in nephritic macrophages (Figure 6C, D). *In vitro* studies showed that *Anxa1* knockdown in LPS-stimulated macrophages resulted in the upregulation of *Fabp4* and downregulation of *Cpt1b* (Figure 6E). This intervention did not have an obvious effect on *Fapb5*. Conversely, treatment with hrANXA1 induced a reduction in *Fabp4* and *Fabp5* mRNA expression and an increase in *Cpt1b* mRNA expression (Figure 6F). In addition, treatment with hrANXA1 significantly reduced Fabp4 expression and mTOR phosphorylation, while co-treatment with the FPR2/ALX antagonist WRW4 abrogated theses effects, confirming FPR2/ALX-dependent regulation of the mTOR/FABP4 axis (Figure 6G). This mechanistic link was further validated by functional experiments in Figure S9, S10. Pharmacological inhibition of Fabp4 in *Anxa1*-deficient macrophages downregulated proinflammatory (*Nos2*, *Tnf-α*) and profibrotic (*Spp1*, *Tgf-β*) gene expression, with reduced Tnf-α and Tgf-β secretion confirmed by ELISA (Figure S11), collectively demonstrating the critical role of Fabp4 in this pathway.

### 3.8 Ac2-26 exerts protective effects against renal injury and fibrosis in MRL/*lpr* mice

**Figure 7:**
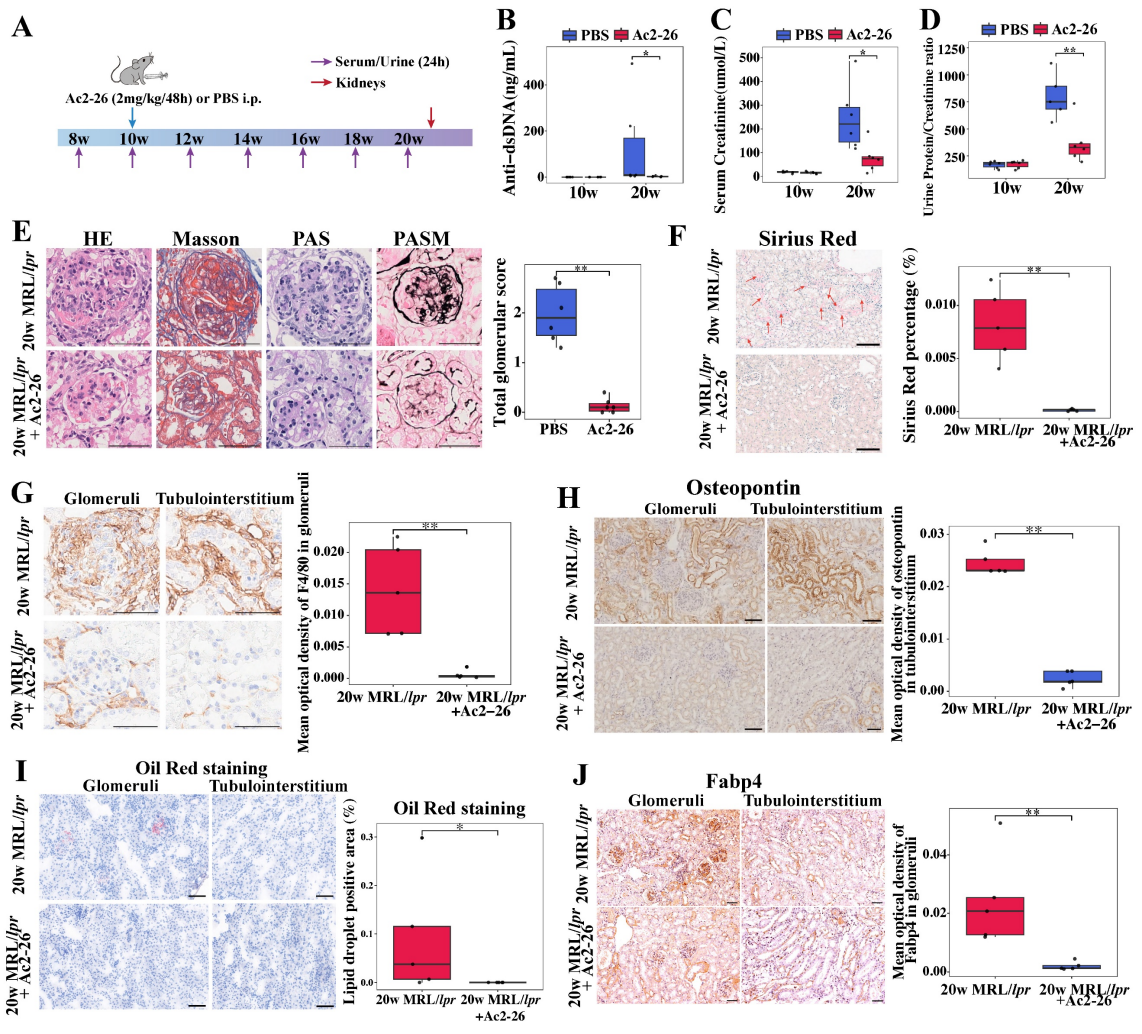
** Ac2-26 administration treats established nephritis in MRL/*lpr* mice.** (A) Ac2-26 treatment protocol for MRL/*lpr* mice. Ac2-26 (2 mg/kg) or PBS treatments were administered i.p. every other day from weeks 10 to 20. n = 6 per group. (B) Levels of anti-dsDNA antibodies in different groups at 10 weeks and 20 weeks (n = 6). (C) Serum creatinine levels in different groups at 10 weeks and 20 weeks (n = 6). (D) Urinary protein/creatinine ratios in different groups at 10 weeks and 20 weeks (n = 6). (E) Representative sections of renal tissue stained with H&E, Masson's trichrome, PAS, and PASM staining and semi-quantitative evaluation of glomerulonephritis at 20 weeks. Bars = 50 μm. (F) Representative photomicrographs and quantitative analysis of Sirius Red staining at 20 weeks. n = 5 per group. Bars = 50 μm. (G) Immunohistochemical staining and quantitative analysis of F4/80 at 20 weeks. n = 5 per group. Bars = 50 μm. (H) Immunohistochemical staining and quantitative analysis of osteopontin (encoded by *Spp1*) at 20 weeks. n = 5 per group. Bars = 50 μm. (I) Representative photomicrographs and quantitative analysis of Oil Red O staining at 20 weeks. n = 5 per group. Bars = 50 μm. (J) Immunohistochemical staining and quantitative analysis of Fabp4 at 20 weeks. n = 5 per group. Bars = 50 μm. Data analyses were performed by Student's *t*-test for two groups. *P < 0.05; **P < 0.01; ***P < 0.001. i.p.: intraperitoneally; HE: hematoxylin-eosin; PAS: periodic acid-Schiff; PASM: periodic acid-silver methenamine.

Based on the observed correlation between elevated renal ANXA1 expression and the expansion of profibrotic* Anxa1^+^Spp1^+^* macrophages during fibrosis progression, we postulated that ANXA1 represents a potential therapeutic target in LN. Given its propensity for dimerization or tetramerization, the synthesized ANXA1 protein may trigger excessive immune responses in animals upon administration, which could adversely affect the therapeutic outcome. Thus, we treated MRL/*lpr* mice during the established phase of nephritis (weeks 10 to 20) with Ac2-26, a synthetic peptide mimicking the bioactive N-terminal domain of ANXA1 (Figure 7A). Ac2-26 administration significantly attenuated lupus-associated autoimmunity and preserved renal function, as shown by reduced serum levels of anti-dsDNA antibodies, lower serum creatinine levels and decreased proteinuria compared with PBS-treated controls (Figure 7B-D). Histopathological evaluation further demonstrated that Ac2-26 mitigated renal injury, with marked reductions in glomerular hypercellularity, mesangial matrix expansion, and glomerular endothelial damage (Figure 7E, based on grading standard in Table S7; more than 10 random glomeruli were counted from each animal). Sirius Red staining confirmed a decrease in renal fibrosis (Figure 7F), and immunohistochemistry revealed significantly reduced macrophage infiltration in Ac-26-treated kidneys (*P* < 0.01; Figure 7G).

At the molecular level, Ac2-26 downregulated the expression of proinflammatory (*Nos2* and *Tnf-α*) and profibrotic (*Spp1* and *Tgf-β*) genes in renal tissue (Figure S12). Consistent with this, osteopontin (encoded by *Spp1*) protein expression was also reduced (Figure 7H). Furthermore, Ac2-26 treatment alleviated renal lipid accumulation, as evidenced by decreased Oil Red O staining (Figure 7I) and lowered renal expression of Fabp4 (Figure 7J). Together, these data indicate that Ac2-26 exerts renoprotective effects in murine LN by attenuating both inflammatory and fibrotic processes, modulating macrophage-driven pathology, and improving lipid metabolic dysregulation.

## 4. Discussion

While the protective effects of ANXA1 have been established in multiple disease contexts, its role in LN remains elusive. Here, our findings demonstrate that elevated renal ANXA1 expression correlates significantly with both fibrotic progression and renal dysfunction in LN patients. Notably, scRNA-seq analysis identified a distinct profibrotic macrophage subset (*Anxa1^+^Spp1^+^*) within nephritic kidneys, paradoxically exhibiting high *Anxa1* expression. Functional studies revealed that ANXA1 promotes macrophage polarization toward an antifibrotic phenotype, potentially through regulating lipid metabolism reprogramming *via* the mTOR/FABP4 signaling pathway. Consequently, therapeutic administration of the ANXA1-mimetic peptide Ac2-26 in MRL/*lpr* mice effectively attenuated disease severity, as evidenced by reduced renal fibrosis and improved renal function.

While emerging data implicate ANXA1 in renal fibrogenesis, its functional contributions exhibit context-dependent duality-exhibiting both pro-resolutive and pro-fibrotic effects across studies 18. Moreover, the molecular mechanisms underlying ANXA1-mediated fibrosis regulation remain undefined, and its pathophysiological role in LN has been insufficiently characterized. Interestingly, Ka et al. reported dense intracellular staining of ANXA1 in the fibrotic region of glomeruli, particularly during crescent formation in the LN 31. In our studies on LN patients, significant associations were observed between kidney ANXA1 expression and the NIH chronicity index, glomerulosclerosis, crescent formation, and interstitial fibrosis—factors known to be associated with renal outcomes and progression to ESRD 11, 32. Additionally, urinary ANXA1 excretion was markedly increased in LN patients and demonstrated significant association with disease severity. However, plasma ANXA1 levels did not differ significantly and showed no correlation with urinary excretion, indicating that kidney tissue origin of ANXA1 in the urine instead of being filtered solely from the blood.

Renal fibrosis pathogenesis is fundamentally shaped by infiltrating immune cells, particularly through their production of pro-fibrotic mediators 9, 33, 34. While ANXA1 is predominantly expressed in myeloid lineages 24, macrophages have emerged as central effectors in both inflammatory and fibrotic renal pathology 10. Moreover, a growing body of research has established a robust correlation between macrophage accumulation within lupus kidneys and disease progression 21, 22. To precisely delineate the role of macrophage-derived ANXA1 in LN, we employed scRNA-seq analysis. One of the most striking findings from our scRNA-seq analysis was the identification and expansion of a previously undescribed subset of monocyte-derived, profibrotic myeloid cells, *Anxa1*^+^*Spp1*^+^ macrophages, in the LN. The prevalence of this subset exhibited dynamic fluctuations throughout the progression of LN. These cells exhibited robust expression of scar-associated genes, including *Spp1*, *Gpnmb*, *Fabp5*, *Trem2*, *Lgals3*, and *CD63*. Notably, while *Trem2* might exert a protective effect against fibrosis, other genes are implicated in fibrosis progression 35-39. These scar-associated gene expression patterns aligned with observations across various fibrotic conditions, characterized by the presence of a specific infiltrating macrophage phenotype known as scar-associated macrophages 40-42. Furthermore, this subset demonstrated significant enrichment of profibrotic processes, including hypoxia response, ECM organization, and fibroblast activation. Pseudotime trajectory and RNA velocity analyses revealed a dynamic transition from anti-inflammatory macrophages to profibrotic *Anxa1*^+^*Spp1*^+^ macrophages, accompanied by a corresponding shift in gene expression programs, indicating their functional plasticity toward a fibrotic phenotype. The functional relevance of this transcriptional profile was further confirmed by gain-of-function experiments, in which *Spp1* overexpression in macrophages directly induced a profibrotic response.

The most important finding of our study was the pronounced expression of *Anxa1* in the infiltrating profibrotic *Anxa1^+^Spp1^+^* macrophages. This was functionally validated by the ANXA1-mimetic peptide Ac2-26 protecting against renal damage by reducing fibrosis, maintaining kidney function, limiting macrophage infiltration and reducing osteopontin (encoded by *Spp1*) expression in MRL/*lpr* mice, while *in vitro* Anxa1 modulation directly regulated key fibrotic markers in macrophages. Together with observed renal ANXA1 upregulation in LN patients, these results establish a causal role for ANXA1 in regulating macrophage fibrogenic potential. We propose that the local upregulation of ANXA1 represents a compensatory mechanism to counteract fibrosis. Thus, the integration of transcriptional profiling, trajectory analysis, functional assays, therapeutic intervention and clinical evidence provides a compelling multi-layered argument for the role of *Anxa1^+^Spp1^+^* macrophage in renal fibrosis.

Previous studies have shown that ANXA1 can direct macrophage polarization, thereby hastening muscle regeneration and protecting against cerebral ischemia-reperfusion injury through the FPR2/ALX-dependent AMPK/mTOR pathway 16, 17. Building upon our previous clinical findings linking mTORC1 activation to LN progression 43 and the therapeutic efficacy of rapamycin in SLE 44, the current study localized mTORC1 hyperactivity to *Anxa1*^+^*Spp1*^+^ macrophages, and established ANXA1-mediated mTOR inhibition. These findings collectively suggest that ANXA1, in addition to mitigating inflammation, specifically inhibits fibrosis by modulating the profibrogenic characteristics of macrophages through mTORC1 signaling.

Notably, an increasing number of studies underscore the close relationship between dysregulated lipid metabolism and renal fibrosis 29, 30. In our scRNA-seq study of *Anxa1^+^Spp1^+^* macrophages, we observed increased signaling indicative of lipid accumulation and reactive oxygen species production, which suggested impaired FAO. Metabolic analysis further revealed significant accumulation of fatty acids in *Anxa1^+^Spp1^+^* macrophages within the nephritic kidneys, which was confirmed by *in vivo* experiments. Fatty acid binding protein 4 (FABP4), a lipid chaperone protein regulated by mTOR signaling 45, 46, was markedly upregulated in *Anxa1^+^Spp1^+^* macrophages during nephritis. Urine levels of FABP4 can serve as a biomarker to distinguish active renal involvement in LN 47. Emerging evidence has established FABP4 as a critical mediator of renal fibrogenesis 48, 49. Our functional studies established ANXA1 as a key regulator of this pathway, with Ac2-26 treatment reducing renal Fabp4 expression in lupus-prone mice. Mechanistically, our findings suggest that ANXA1 may counteract renal fibrosis by modulating the mTOR/FABP4 signaling pathway, improving FAO, and thus driving the reprogramming of macrophages toward an antifibrotic phenotype and reducing lipid accumulation in macrophages in the context of LN.

In summary, our findings demonstrate a significant correlation between elevated renal ANXA1 expression and fibrotic progression in human LN. scRNA-seq analysis further elucidated monocyte/macrophage heterogeneity, identifying a unique profibrotic *Anxa1^+^Spp1^+^* macrophage subset with elevated *Anxa1* expression in nephritic kidneys. Mechanistically, ANXA1 exerts antifibrotic effects through modulation of lipid metabolism *via* inhibition of the mTOR/FABP4 signaling pathway. Pharmacological intervention with the ANXA1 mimetic peptide Ac2-26 effectively attenuates renal injury in murine LN models. These discoveries collectively establish a robust rationale for developing ANXA1-based therapeutics targeting the mTOR/FABP4 axis as a novel therapeutic strategy for managing LN progression.

Several limitations warrant careful consideration when interpreting the findings of this study. Firstly, a methodological limitation lies in the absence of direct *in vivo* evidence confirming the profibrotic capacity of *Anxa1^+^Spp1^+^* macrophages. To definitively establish their pathogenic role, future investigations employing conditional knockout models in conjunction with temporally-regulated macrophage depletion strategies would be essential. Secondly, while our data suggest that infiltrating monocytes serve as the primary precursors of *Anxa1^+^Spp1^+^* macrophages, this conclusion remains inferential. Definitive determination will require advanced lineage-tracing methodologies, such as inducible genetic fate-mapping systems or parabiosis experiments, to conclusively substantiate this cellular origin.

## Supplementary Material

Supplementary methods, figures and tables 2-3, 5-7.

Supplementary table 1.

Supplementary table 4.

## Figures and Tables

**Table 1:** Association between glomerular ANXA1 expression and clinicopathological features in patients with lupus nephritis (n = 33).

Glomerular ANXA1 expression		
Clinical features		*P* value
Hypertension (No/Yes)	0.003 (0.001, 0.029)/0.011 (0.002, 0.031)	0.316
NS (No/Yes)	0.021 (0.002, 0.059)/0.007 (0.001, 0.027)	0.302
AKI (No/Yes)	0.003 (0.001, 0.020)/0.021 (0.003, 0.051)	0.048
Hematuria^1^(No/Yes)	0.003 (0, 0.044)/0.011 (0.001, 0.030)	0.567
Leukocyturia (noninfectious)^2^(No/Yes)	0.019 (0.002, 0.035)/0.003 (0.001, 0.009)	0.047
	r value	*P* value
Age (years)	0.428	0.013
SLEADI	-0.151	0.403
Hb (g/L)	-0.007	0.971
Urinary protein (g/day)	0.178	0.347
Serum creatinine (μmol/L)	0.970	0.592
C3 level (g/L)	0.068	0.709
C4 level (g/L)	-0.079	0.661
Anti-dsDNA antibodies	-0.121	0.533
Anti-C1q antibodies	-0.474	0.008
Renal histopathologic features (light microscopy)		*P* value
Histologic classes (nonproliferative/proliferative)	0 (0, 0.003)/0.011 (0.001, 0.032)	0.045
Neutrophils exudation/karyorrhexis (No/Yes)	0.003 (0, 0.028)/0.009 (0.001, 0.030)	0.448
Fibrinoid necrosis (No/Yes)	0.009 (0.001, 0.033)/0.007 (0.001, 0.018)	0.385
Mesangial hypercellularity (No/Yes)	0.002 (0.001, 0.021)/0.013 (0.001, 0.034)	0.235
Endocapillary hypercellularity (No/Yes)	0.004 (0.001, 0.028)/0.013 (0.001, 0.043)	0.377
Hyaline deposits (No/Yes)	0.016 (0, 0.043)/0.004 (0.001, 0.020)	0.602
	r value	*P* value
AI	0.023	0.921
Cellular/fibrocellular crescents	0.359	0.043
Interstitial inflammation	0.168	0.349
CI	0.548	0.010
Glomerulosclerosis	0.576	<0.001
Tubular atrophy	0.202	0.260
Interstitial fibrosis	0.373	0.033
Tubulointerstitial ANXA1 expression	0.942	<0.001
Renal histopathologic features (direct immunofluorescence)		*P* value
IgG deposition (≤2+/>2+)	0.003 (0.001, 0.034)/0.010 (0.001, 0.027)	0.773
IgA deposition (≤2+/>2+)	0.008 (0.001, 0.024)/0.009 (0.002, 0.034)	0.373
C3c deposition (≤2+/>2+)	0.010 (0.001, 0.022)/0.007 (0.001, 0.031)	0.821
C1q deposition (≤2+/>2+)	0.012 (0.001, 0.034)/0.004 (0.001, 0.016)	0.506

Notes: ^1^Defined as red blood cell count ≥ 5/HPF, ^2^Defined as WBC ≥ 5/HPF. NS: nephrotic syndrome; AKI: acute kidney injury, is defined as any of the following criteria on the basis of the KDIGO criteria: an increase in serum creatinine of × 0.3 mg/dl (× 26.5 µmol/L) within 48 hours or an increase in serum creatinine to × 1.5 times baseline, which is known or presumed to have occurred within the previous 7 days, or a urine volume < 0.5 ml/kg per hour for 6 hours; SLEDAI: systemic lupus erythematosus disease activity index; Hb: hemoglobin; C3: complement component 3; C4: complement component 4; dsDNA: double-stranded DNA; NIH: National Institutes of Health; AI: NIH activity index; CI: NIH chronicity index. Data analyses were performed by Mann-Whitney *U* test for two groups. Correlations were carried out *via* the Spearman test. Data expressed as median (interquartile ranges).

## Data Availability

The data supporting the findings of this study are openly available in the BIG Sub database under accession number CRA037374. Requests for further access to datasets can be directed to the corresponding author.

## Abbreviations

LN: lupus nephritis
ANXA1: Annexin A1
SLE: Systemic lupus erythematosus
ESRD: end-stage renal disease
CKD: chronic kidney disease
scRNA-seq: single-cell RNA sequencing
FAO: fatty acid oxidation
USTC: the University of Science and Technology of China
PCR: polymerase chain reaction
eGFR: estimated glomerular filtration rate
FACS: fluorescence-activated cell sorting
UMAP: uniform manifold approximation and projection
GSEA: gene set enrichment analysis
TLR: Toll-like receptor
ECM: extracellular matrix
mTORC1: the mammalian target of rapamycin complex 1
FABP4: fatty acid binding protein 4
