# Supplementary material for: ANXA1-mediated mTOR/FABP4 Inhibition Drives Antifibrotic Macrophage Reprogramming in Lupus Nephritis
Source: Int J Biol Sci. 2026 Feb 4;22(5):2343–60. doi: 10.7150/ijbs.118613 (PMC12965141; doi:10.7150/ijbs.118613)
Supplement: Supplementary file 1 — Supplementary methods, figures and tables 2-3, 5-7. [file ijbsv22p2343s1.pdf]

Supplementary Material

ANXA1-mediated mTOR/FABP4 inhibition drives antifibrotic macrophage reprogramming in lupus nephritis

Juan Tao, Qingyu Cheng, Pinjie Zhang, Guizhen Yu, Qi Chen, Manwen Yang, Qiqin Wu, Haopeng Fang, Haibo Wu, Xiaoyuan Song, Zhu Chen, Min Chen, Xiaoming Meng, Mingxing Lei, Tengchuan Jin

This file includes:

1. **Supplementary Methods**
2. **Supplementary Figures**
3. **Supplementary Tables**

1. Supplementary Methods

Human subjects

Thirty-three patients who were hospitalized at the First Affiliated Hospital of the University of Science and Technology of China (USTC) from January 2018 to September 2022 and who underwent renal biopsy with a pathological diagnosis of only lupus nephritis (LN) were included in this study. All patients with SLE were diagnosed according to the revised criteria of the 1997 American College of Rheumatology [1]. The clinical characteristics of the enrolled LN patients at the time of kidney biopsy, including data on age, sex, urinary total protein, serum creatinine, eGFR (according to the Chronic Kidney Disease Epidemiology Collaboration [CKD-EPI] equation), serum C3 and C4, and the Systemic Lupus Erythematosus Disease Activity Index (SLEDAI) score, are summarized in Table S2. LN was reclassified in accordance with the 2003 International Society of Nephrology/Renal Pathology Society pathologic classification system [2]. Kidney biopsy samples were collected to measure ANXA1 protein expression in the kidney.

Urine and plasma samples from another 36 patients with LN and 41 nonrenal SLE patients [3] were collected for measuring urinary and plasma ANXA1 levels, respectively. Urine and plasma samples from healthy controls were obtained from ethically matched volunteers. The human studies followed the Declaration of Helsinki, and the design of this work was approved by local ethical committees [the First Affiliated Hospital of USTC, approval number: 2023KY130]. Informed consent was obtained from all participants.

Histological analysis

Paraffin-embedded kidney sections (4 μm) were subjected to hematoxylin-eosin (HE), Masson's trichrome, periodic acid–Schiff (PAS), and periodic acid–silver methenamine (PASM) staining. Based on PAS staining, glomerulonephritis was graded on a semiquantitative scale as described previously [4, 5]. The grading standards are listed in Table S4. Ten random glomeruli were counted from each mouse.

Immunohistochemistry and immunofluorescence

Paraffin-embedded renal sections were subjected to heating in citrate buffer in a microwave for antigen retrieval. For immunohistochemistry, the tissues were blocked with peroxidase-blocking buffer for 10 min at 37 °C and 3% BSA for 30 min at 37 °C and then incubated with primary antibodies (anti-ANXA1, anti-osteopontin, and anti-FABP4) at 4 °C overnight. Subsequently, secondary antibodies were applied, and detection was performed with DAB. Nuclei were counterstained with hematoxylin solution. All images were acquired *via* the same microscope and camera set. The intensity of specific immunohistochemical staining was measured *via* Image-Pro Plus software (Media Cybernetics). The intensities of the positive staining were determined *via* the mean integrated optical density (mean IOD) per area of tissue (400× magnification). All glomeruli in each kidney section were analyzed for human kidney tissues. The glomerular area was measured by tracing around the perimeter of the glomerular tuft. Correlation studies were carried out between ANXA1 expression and clinical and pathological parameters.

The primary antibodies used for immunofluorescence included anti-ANXA1 and anti-CD68. The primary antibodies were incubated with the tissues overnight at 4 °C, followed by incubation with fluorophore-conjugated secondary antibodies. Images of random visual fields were acquired on an optical microscope (Olympus) and Zeiss LSM 780 confocal microscope (Carl Zeiss). The antibodies used for immunohistochemistry and immunofluorescence are listed among the key resources.

Blood and Urine Examination

The concentration of ANXA1 in the urine and plasma samples was measured *via* enzyme-linked immunosorbent assay (ELISA) *via* human ANXA1 ELISA kits (Abcam) according to the manufacturer's instructions. The urine creatinine level was measured with a creatinine test kit (Nanjing Jiancheng Bioengineering Institute) according to the manufacturer's instructions.

The levels of anti-dsDNA antibodies in the serum were determined *via* an anti-dsDNA ELISA kit (EK20313, Signalway Antibody) according to the manufacturer's recommendations. The 24-h urine protein and urine creatinine concentrations in MRL/*lpr* mice were measured *via* a protein quantification test kit (C035-2-1; Nanjing Jiancheng Bioengineering Institute) and a creatinine test kit (C011-2-1; Nanjing Jiancheng Bioengineering Institute), respectively. The levels of serum creatinine were also tested *via* a creatinine test kit (C011-2-1; Nanjing Jiancheng Bioengineering Institute). Assays were performed according to commercial kits and the manufacturer's instructions (Nanjing Jiancheng Bioengineering Institute).

Preparation of single peripheral blood mononuclear cells

The mice were anesthetized, and 0.6-1.0 ml of whole blood was collected through the right atrium of the heart. Red blood cell lysis buffer (Invitrogen) was added while the samples were maintained on ice for 5 mn, and the samples were subsequently centrifuged at 400 × g and 4 °C for 5 mn. The cells were then washed twice in prechilled PBS supplemented with 2% FBS. The final step involved another centrifugation step, followed by resuspension of the cells in prechilled PBS containing 2% FBS.

Preparation of single cells from the kidney

The mice were anesthetized and perfused with prechilled PBS *via* the left heart ventricle. The kidneys were subsequently collected and immersed in cold 1640 medium, after which the visceral fat and kidney capsule were excised. The kidneys were then sectioned into 1 mm^3 pieces with small scissors on ice, followed by incubation in 5 ml of digestion buffer containing 1 mg/ml collagenase type I (Sigma-Aldrich) and 50 U/ml DNase I (NEB) at 37 °C for 30 mn with agitation. The enzymatic digestion was stopped by the addition of 5% FBS. The digested tissue was then strained through a 70 μm cell strainer into prechilled PBS supplemented with 2% FBS on ice. The cells were pelleted by centrifugation at 400 × g and 4 °C for 5 mn. The cell pellet was further processed by incubation with red blood cell lysis buffer on ice for 5 mn, followed by another centrifugation. The cells were then washed with prechilled PBS containing 2% FBS. Finally, the cells were centrifuged and

resuspended in prechilled PBS containing 2% FBS.

Fluorescence-activated cell sorting (FACS) for single-cell RNA sequencing

Single-cell suspensions were stained in cell staining buffer with fluorochrome-labeled antibodies against F4/80, Cd11b, Ly6c, Cd45, and 7-AAD. The cells were subsequently sorted *via* an Aria sorp and a cell sorter (BD Biosciences). Flow cytometry parameters, including FSC and SSC, were employed to exclude cell debris, whereas FSC A and W parameters were employed to exclude cell adhesion. The inclusion of 7-AAD facilitated the exclusion of dead cells, thereby increasing the viability of the cells. Next, the $Cd45^{+}$ cells were sorted, and subsequently, the resulting cells for each sample were identified on the basis of representative markers. To obtain enough monocytes/macrophages, both $Cd11b^{+}$ and $F4/80^{+}$ cells were sorted from kidney samples, whereas $Cd11b^{+}Ly6c^{+}$ cells were sorted from blood samples. The detailed gating strategies are depicted in Figure 3C.

Quality control, dimensionality reduction and clustering of single-cell RNA sequencing data

scRNA-seq data processing was conducted *via* Seurat (v4.3) [6]. Cells with a mitochondrial content exceeding 30% were excluded from the analysis. Multisample integration was performed with the Harmony algorithm [7]. The normalization and scaling of gene expression were subsequently performed *via* the NormalizeData and ScaleData functions in Seurat [6]. Dimensionality reduction was performed *via* the

RunPCA and RunUMAP functions, and clustering analysis was performed *via* the FindClusters function in Seurat [6]. Finally, the uniform manifold approximation and projection algorithm was employed to visualize the cells in a two-dimensional space.

Differential expression analysis

To identify the differentially expressed genes (DEGs), the FindAllMarkers function was employed to conduct a likelihood ratio test. The criteria for gene selection included those that were expressed in at least 10% of the cells within a cluster and exhibited an average log-fold change greater than 0.25.

Pathway enrichment analysis

To identify the potential functional roles of the DEGs, we performed Kyoto Encyclopedia of Genes and Genomes (KEGG) enrichment analysis *via* the clusterProfiler package (version 3.16.1) [8]. Pathways with an adjusted *P* value less than 0.05 were considered to be significant. For the gene set enrichment analysis (GSEA), the hallmark, Reactome, Gene Ontology (GO), and KEGG pathways, as curated in the MsigDB [9] were referenced.

RNA velocity analysis and trajectory construction

The Velocyto [10] package was used to obtain the spliced and unspliced count matrix, and the standard pipeline of the scVelo [11] python package was used to calculate and visualize the RNA velocity of the scRNA-seq data. Briefly, the

"min_shared_counts" parameter was set to 20, and "n_top_genes" was set to 2000 in the "pp.filter_and_normalize ()" function for preliminary filtering and normalization of the spliced and unspliced RNA expression matrices. PCA-based dimensional reduction of the two matrices was performed *via* the "pp.moments ()" function, with the number of neighboring cells set to 30. The functions "tl.velocity ()" and "tl.velocity_graph ()" were executed with default parameters to calculate the RNA velocity, and the "pl.velocity_embedding_stream ()" function was used to visualize the RNA velocity stream on the UMAP graph.

Single-cell pseudotime trajectories of monocyte/macrophage populations were reconstructed *via* the Monocle 3 (v1.3.1) [12] package. Following data preprocessing with the preprocess_cds function, batch effects were removed *via* the "align_cds" function [13]. The "learn_graph" and "order_cells" functions were subsequently employed to generate trajectories and order cells along pseudotime. Considering the RNA velocity stream, Cluster 3 and Cluster 6 were set as root nodes for trajectory analysis.

Cell culture

RAW264.7 macrophages were cultured and transfected with concentration-matched pairs of scrambled or gene-targeted small interfering RNAs (*Anxa1* siRNAs; GenePharma Co., Ltd.) for 6 h *via* the GP-transfect-Mate system (GenePharma) according to the manufacturer's instructions. To induce inflammatory responses, the cells were exposed to one of the Toll-like receptor ligands, LPS (10

ng/ml; Sigma-Aldrich). To investigate the function of ANXA1 in RAW264.7 macrophages, the cells were stimulated with LPS (10 ng/ml) for 24 h, followed by treatment with human recombinant ANXA1 (10 nM, R&D Systems) in the presence or absence of WRW4 (10 μM, GLPBIO), a compound known to antagonize FPR2/ALX [14].

The *Spp1* overexpression and control lentiviral vectors were constructed by Hefei Juyan Biotechnology Co., Ltd. The *Spp1*-overexpression (Spp1-OE), and control (Spp1-NC) were generated by lentiviral transduction according to the manufacturer's protocol. The cells with stable expression were identified by screening with culture medium supplemented with puromycin (Yeasen) at a final concentration of 10 μg/mL after 2 weeks. The sequences used for the overexpression of Spp1 are listed in Table S1.

To generate bone marrow-derived macrophages (BMDMs), bone marrow cells were isolated from the femurs and tibias of female C57BL/6J mice (6-8 weeks old). After erythrocyte lysis, cells were differentiated for 7 days in DMEM supplemented with 10% FBS, 1% penicillin/streptomycin, and 15% L-929 cell-conditioned supernatant (as an M-CSF source), with a medium change on day 3. The resulting BMDMs were then stimulated as indicated, and culture supernatants were collected for ELISA.

Anxa1 knockout mice were generated by Shanghai Model Organisms Center, Inc. (SMOC). BMDMs were isolated and treated with the FABP4 inhibitor BMS-309403 (10 μM, MedChemExpress).

Quantitative real-time polymerase chain reaction (PCR) analysis

A Hipure Total RNA Mini Kit (Magen) was used to extract total RNA from cultured cells or mouse kidney tissues. cDNA was subsequently synthesized from 1 μg of RNA *via* a high-capacity cDNA reverse transcription kit (TaKaRa). Quantitative real-time PCR was performed *via* a Lightcycler 96 PCR system (Roche) with TB Green reagent (TaKaRa). The PCRs were executed for 40 cycles. The mRNA expression levels of each target gene were normalized to those of GAPDH, and the relative mRNA expression levels in the experimental group were compared with those in the control groups *via* the -ΔΔCt method. The primers used for mRNA detection are listed in Table S1.

Western blot analysis

Proteins were extracted from RAW264.7 macrophages. Equal amounts of protein were separated *via* sodium dodecyl sulfate-polyacrylamide gel electrophoresis and transferred to a polyvinylidene fluoride membrane. The following proteins were subsequently detected with appropriate antibodies: FABP4 (Abcam) and phospho-mTOR (Ser2448; Cell Signaling Technology). The band intensity was quantified with ImageJ software. The antibodies used for western blotting are listed among the key resources.

To investigate the role of AMPK in the ANXA1-FPR2/ALX axis-mediated regulation of the mTOR/FABP4 signaling pathway, the AMPK inhibitor Compound C (HY-13418A; MedChemExpress) was used. A 10mM stock solution was prepared by

dissolving Compound C in dimethyl sulfoxide (DMSO) and stored at -20°C. BMDMs were isolated and pre-treated with Compound C at a final concentration of 10 μM for 1 h, followed by treatment with human recombinant ANXA1 (10 nM, R&D Systems). Cells were ultimately harvested for subsequent Western blot analysis.

Co-immunoprecipitation (Co-IP)

Cells were lysed and the protein concentrations were measured. For co-IP, lysates was incubated with 2-20 μg anti-FPR2, anti-ANXA1, anti-AMPK-α or anti-IgG antibodies coated on beads on a rotator (room temperature, 30 mn; 4 °C, 2 h). The beads were washed to remove non-bound material and eluted in a low-pH elution buffer that could dissociated bound antigen from the antibody-crosslinked beads. The precipitate was separated by SDS-PAGE and detected by immunoblotting. IgG was used as a negative control.

Condensed Methods for FPR2-AMPK: Structure, Docking, and MD simulation

Amino acid sequences of FPR2 (UniProt ID: P25090) and AMPK (UniProt ID: Q9Y478) were retrieved from the UniProt database. Their three-dimensional (3D) structures were predicted using AlphaFold 3 and optimized via Schrödinger's Protein Preparation Wizard, which included supplementing missing side-chain atoms, optimizing hydrogen bond (H-bond) networks, assigning protonation states, and performing restrained energy minimization under the OPLS4 force field to eliminate atomic clashes. Prepared FPR2 and AMPK structures were imported into

Schrödinger's protein-protein docking module. After docking sampling, the optimal binding conformation (docking score: -788.27; binding energy: -1168.78) was selected using a comprehensive scoring function. LigPlot+ was used to analyze H-bond interactions at the binding interface, and PyMOL for structural visualization. To evaluate the dynamic stability of the FPR2-AMPK complex, molecular dynamics (MD) simulation was conducted using the GROMACS software package with the CHARMM36 force field: the complex was placed in a cubic water box, and ions were added to neutralize the system and mimic physiological ion concentration. The system first underwent energy minimization, followed by equilibration under the NVT and NPT ensembles, and finally a 100 ns production run. Trajectory analysis was performed using the last 80 ns of stable data, with calculations of root mean square deviation (RMSD), root mean square fluctuation (RMSF), radius of gyration (Rg), solvent-accessible surface area (SASA), and the number of intermolecular H-bonds. The free energy landscape was constructed via the GMX_MMPBSA tool and self-written scripts base on RMSD and Rg data.

References

1. Hochberg MC. Updating the American College of Rheumatology revised criteria for the classification of systemic lupus erythematosus. Arthritis Rheum. 1997; 40: 1725.
2. Weening JJ, D'Agati VD, Schwartz MM, et al. The classification of glomerulonephritis in systemic lupus erythematosus revisited. J Am Soc Nephrol. 2004; 15: 241-50.
3. Li Y, Tang C, Vanarsa K, et al. Proximity extension assay proteomics and renal single cell transcriptomics uncover novel urinary biomarkers for active lupus nephritis. J Autoimmun. 2024; 143: 103165.
4. Zhou M, Guo C, Li X, et al. JAK/STAT signaling controls the fate of CD8(+)CD103(+) tissue-resident memory T cell in lupus nephritis. J Autoimmun. 2020; 109: 102424.
5. Wang H, Shen M, Ma Y, et al. Novel mitophagy inducer alleviates lupus nephritis by reducing myeloid cell activation and autoantigen presentation. Kidney Int. 2024; 105: 759-74.
6. Hao Y, Hao S, Andersen-Nissen E, et al. Integrated analysis of multimodal single-cell data. Cell. 2021; 184: 3573-87.
7. Korsunsky I, Millard N, Fan J, et al. Fast, sensitive and accurate integration of single-cell data with Harmony. Nat Methods. 2019; 16: 1289-96.
8. Yu G, Wang LG, Han Y, et al. clusterProfiler: an R package for comparing biological themes among gene clusters. Omics. 2012; 16: 284-7.
9. Bhuva D, Smyth G, Garnham A. msigdb: An ExperimentHub package for the molecular signatures database (MSigDB), R package version. 2023; 1.
10. La Manno G, Soldatov R, Zeisel A, et al. RNA velocity of single cells. Nature. 2018; 560: 494-98.
11. Bergen V, Lange M, Peidli S, et al. Generalizing RNA velocity to transient cell states through dynamical modeling. Nat Biotechnol. 2020; 38: 1408-14.
12. Cao J, Spielmann M, Qiu X, et al. The single-cell transcriptional landscape of mammalian organogenesis. Nature. 2019; 566:496-502.
13. Haghverdi L, Lun ATL, Morgan MD, et al. Batch effects in single-cell RNA-sequencing data are corrected by matching mutual nearest neighbors. Nat Biotechnol. 2018; 36: 421-27.
14. Senchenkova EY, Ansari J, Becker F, et al. Novel Role for the AnxA1-Fpr2/ALX Signaling Axis as a Key Regulator of Platelet Function to Promote Resolution of Inflammation. Circulation. 2019; 140: 319-35.

2. Supplementary Figures

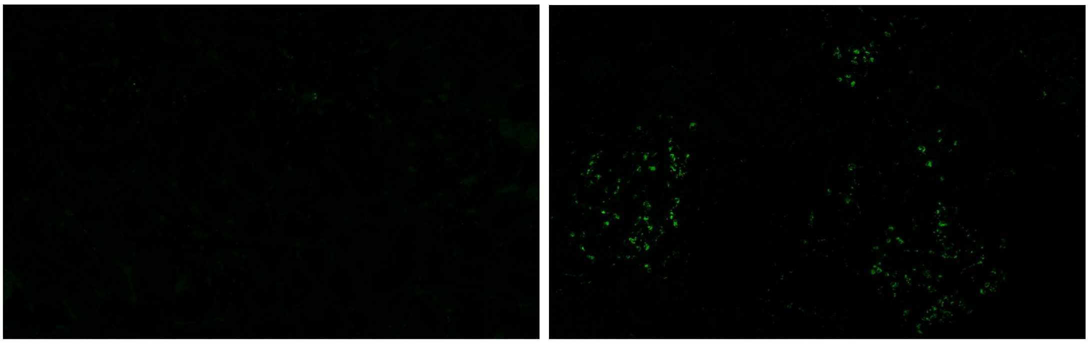

Figure S1. Representative images of CD68 staining in human renal cortical tissue from lupus nephritis and non-neoplastic adjacent control tissue.

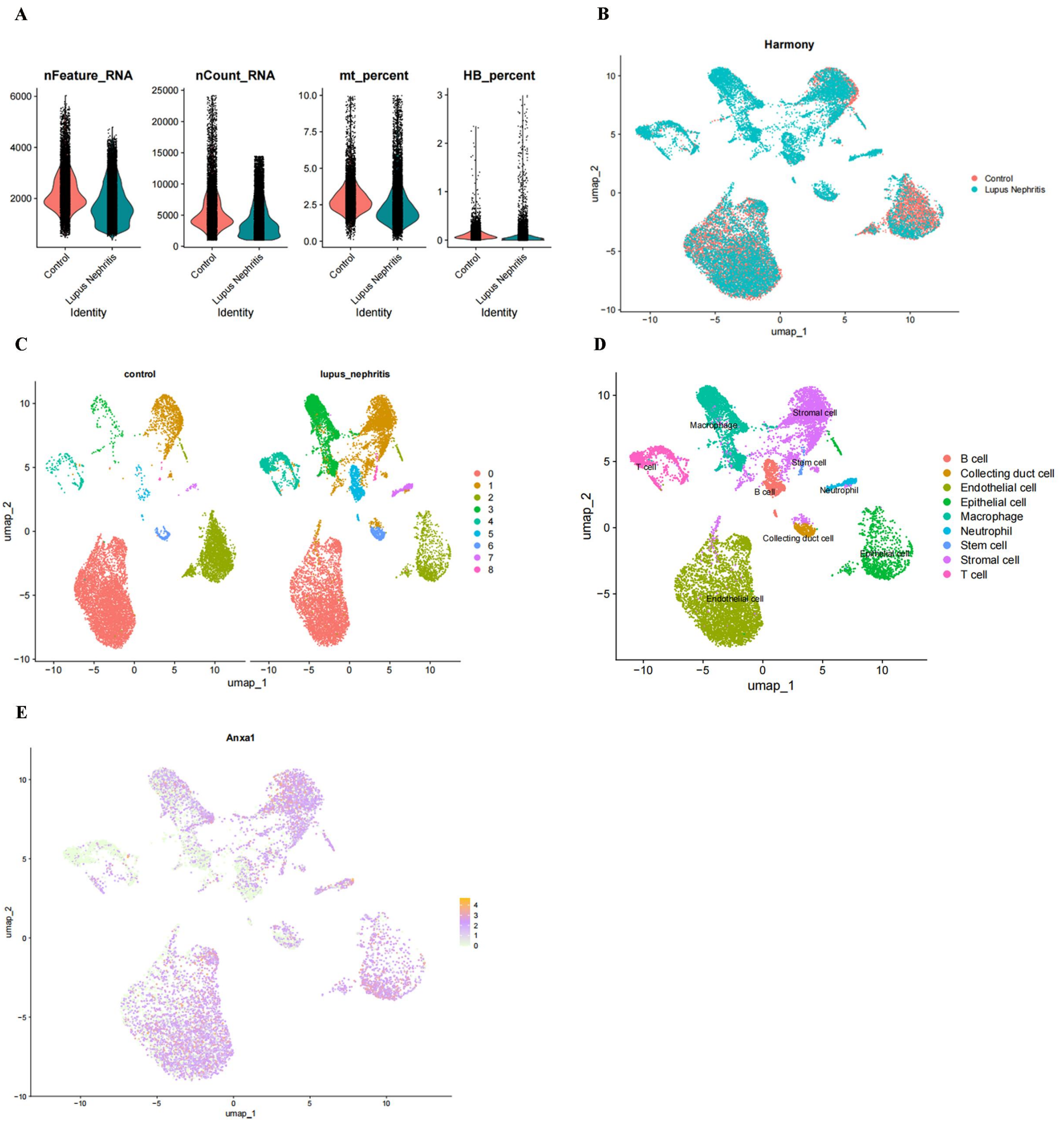

Figure S2. Single-cell RNA sequencing analysis of lupus nephritis: quality control, cell annotation and *Anxa1* expression.

(A) Violin plots for quality control of all data. **(B)** Uniform manifold approximation and projection plot colored according to cell clusters, depicting cell annotation. **(C)** Uniform manifold approximation and projection plots illustrate the distribution of cell clusters across the control and lupus nephritis groups. **(D)** Cell cluster identities were annotated based on statistically expression of established lineage-specific markers. **(E)** Uniform manifold approximation and projection plots demonstrating the expression of *Anxa1* in distinct subsets.

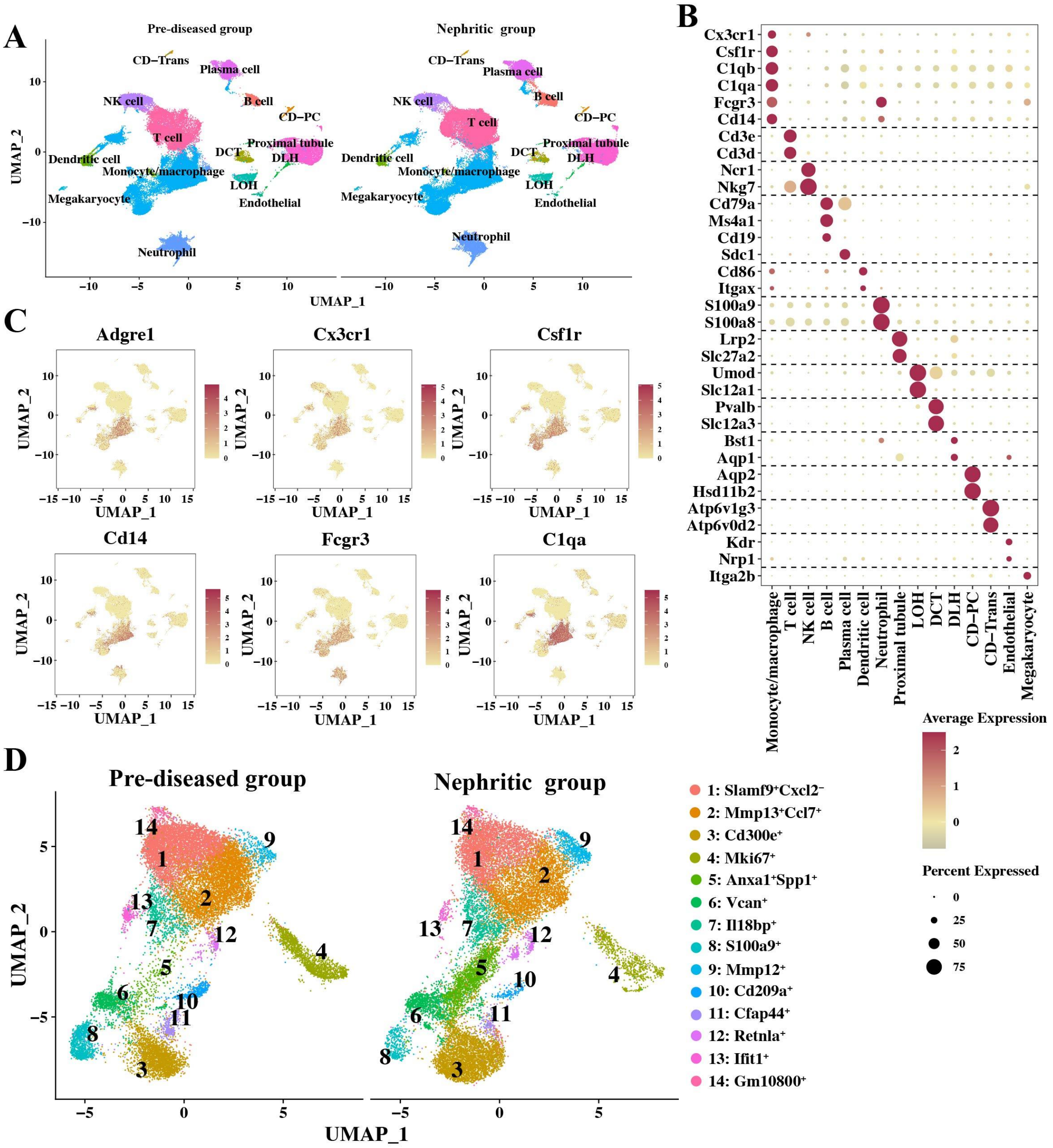

Figure S3. Identification of monocyte/macrophage subpopulations in single-cell RNA sequencing data of sorted cells from the kidney and blood.

(A) Uniform manifold approximation and projection visualization of a total of 116,950 cells identified 15 different clusters after unsupervised clustering, including 55,676 pre-diseased and 61,274 nephritic cells. Each point depicts a single cell, which is colored according to cluster designation. (**B)** Dot plot displaying the representative marker genes in each cell type. (**C)** Uniform manifold approximation and projection plots demonstrating representative monocyte/macrophage markers. (**D)** Uniform manifold approximation and projection plots demonstrating the monocyte/macrophage cluster distribution at different stages.

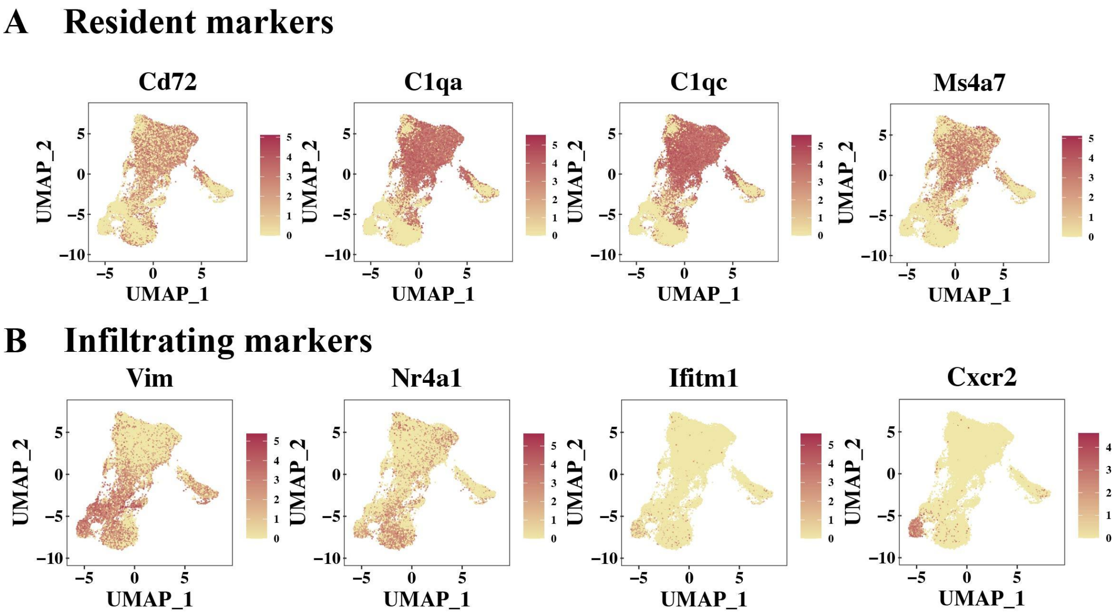

Figure S4. Uniform manifold approximation and projection plots demonstrating representative markers.

Markers representing resident **(A)** and infiltrating **(B)** macrophages.

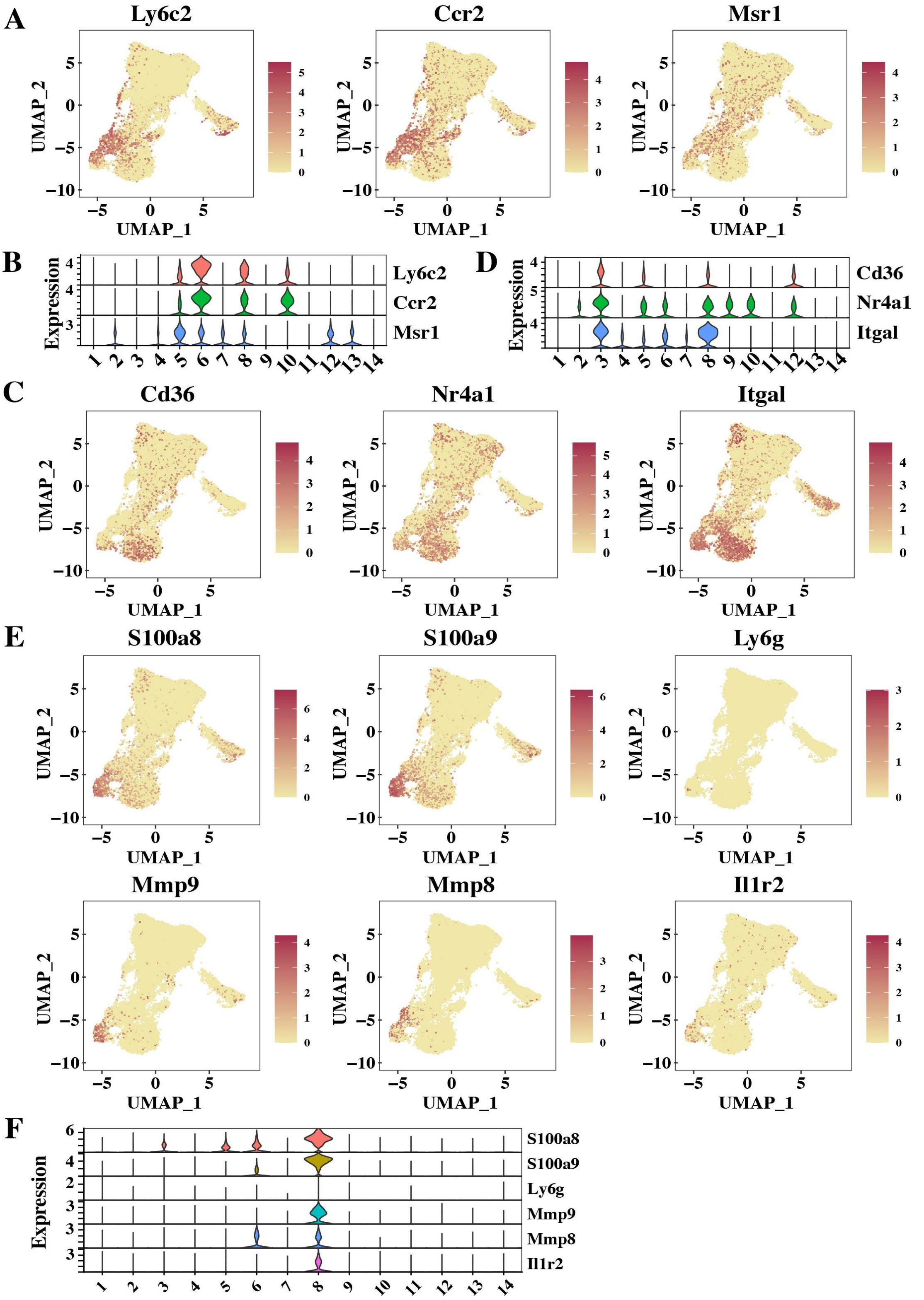

Figure S5. Representative marker gene expression within the infiltrating monocyte/macrophage subpopulations.

Uniform manifold approximation and projection plots **(A)** and violin plots **(B)** demonstrate the representative markers in the infiltrating monocyte/macrophage subpopulations. Uniform manifold approximation and projection plots **(C)** and violin plots **(D)** demonstrate the key markers in Cluster 3. Uniform manifold approximation and projection plots **(E)** and violin plots **(F)** demonstrate the key markers in Cluster 8.

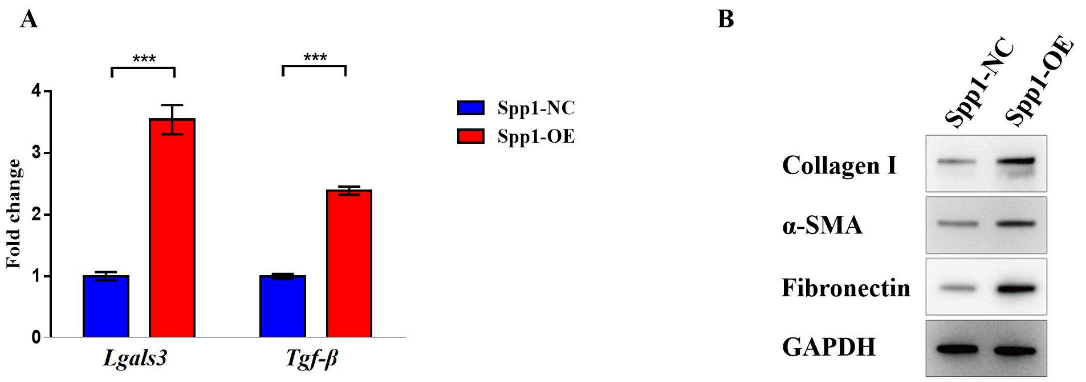

Figure S6. ***Spp1*** **overexpression promotes a profibrotic phenotype in macrophages.**
(**A)** Relative mRNA levels of the profibrotic genes *Lgals3* (encoding galectin-3) and *Tgf-β* in RAW264.7 macrophages transfected with a *Spp1* overexpression construct (Spp1-OE) versus negative control (Spp1-NC). n = 3 per group. Data analyses were performed by Student's *t*-test for two groups. ***$P < 0.001$. (**B)** Representative western blot analysis validating the upregulation of the extracellular matrix proteins collagen I, fibronectin, and the myofibroblast marker α-SMA at the protein level in Spp1-OE macrophages. Spp1-NC: Spp1-NC RAW264.7 macrophages; Spp1-OE: Spp1-overexpressing RAW264.7 macrophages.

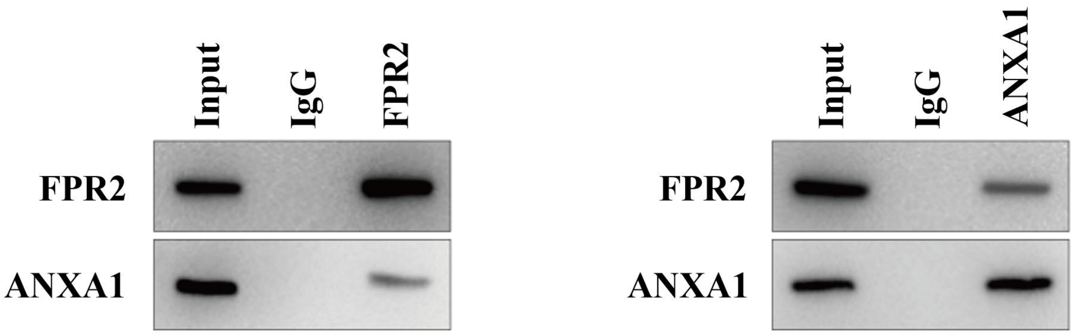

Figure S7. Co-immunoprecipitation assays showing the interaction between ANXA1 and FPR2. The left panel demonstrates that ANXA1 is co-immunoprecipitated with FPR2 using an anti-FPR2 antibody. The right panel shows that FPR2 is co-immunoprecipitated with ANXA1 using an anti-ANXA1 antibody. IgG serves as a negative control, and Input represents the total protein lysate.

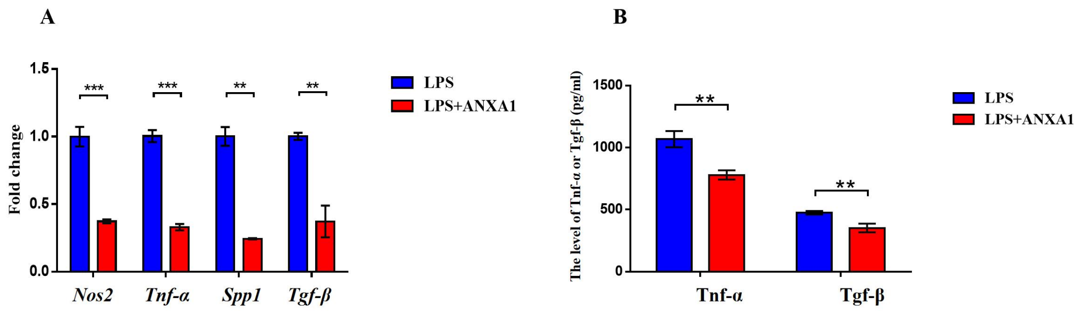

Figure S8. The role of Anxa1 on macrophage polarization using BMDMs.

(A) Quantitative real-time polymerase chain reaction analysis of proinflammatory (*Nos2*, *Tnf-α*) and profibrotic (*Spp1*, *Tgf-β*) gene expression in BMDMs treated with 10 nM human recombinant ANXA1 for 24 h under LPS stimulation. n = 3 per group. **(B)** ELISA quantifying the secretion of Tnf-α and Tgf-β. n = 3 per group. Data analyses were performed by Student's *t*-test for two groups. $^{**}P < 0.01$; $^{***}P < 0.001$.

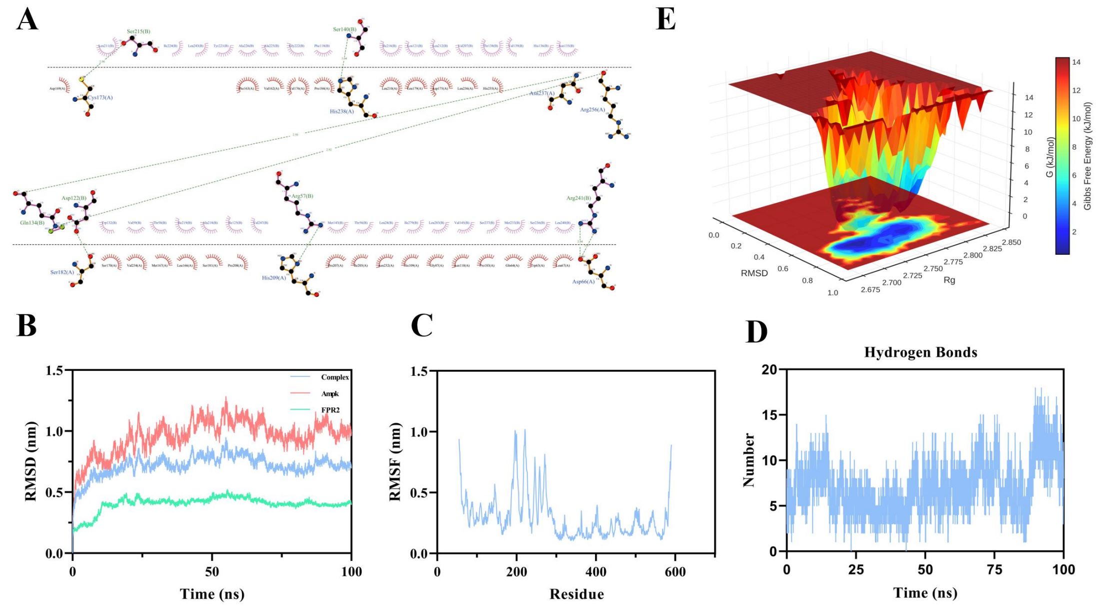

Figure S9. Molecular dynamics simulation analysis of the AMPK-FPR2 complex.

(A) Binding conformation of FPR2 and AMPK. The optimal conformation exhibits a binding score of -788.27 and binding energy of -1168.78. Interaction analysis reveals 8 hydrogen bonds formed between FPR2 residues (Ser215, Ser140, Gln134, Asp122, Arg57, and Arg241) and AMPK residues (Cys173, His238, Asn237, Arg256, Ser182, His209, and Asp66), indicating a strong and specific interaction. **(B)** RMSD profile of the FPR2-AMPK complex during 100 ns molecular dynamics simulation. The complex reaches equilibrium at ~20 ns and remains stable for the subsequent 80 ns, with RMSD fluctuations < 0.2 nm, confirming no drastic structural changes and sustained conformational stability in the solvent environment. **(C)** RMSF analysis of FPR2 and AMPK in the complex state. Both proteins show generally low RMSF values (most < 0.5 nm), particularly in the binding interface region, indicating reduced overall flexibility and increased structural rigidity post-binding. **(D)** Hydrogen bond count during 100 ns MD simulation. The average number of hydrogen bonds is 7.04 (maximum = 18), demonstrating that hydrogen bond interactions are not only maintained but also highly abundant under dynamic conditions, serving as a key driving force for complex stability. **(E)** Gibbs free energy landscape plotted against RMSD and radius of gyration (Rg), with blue regions representing low-energy stable conformations of the complex.

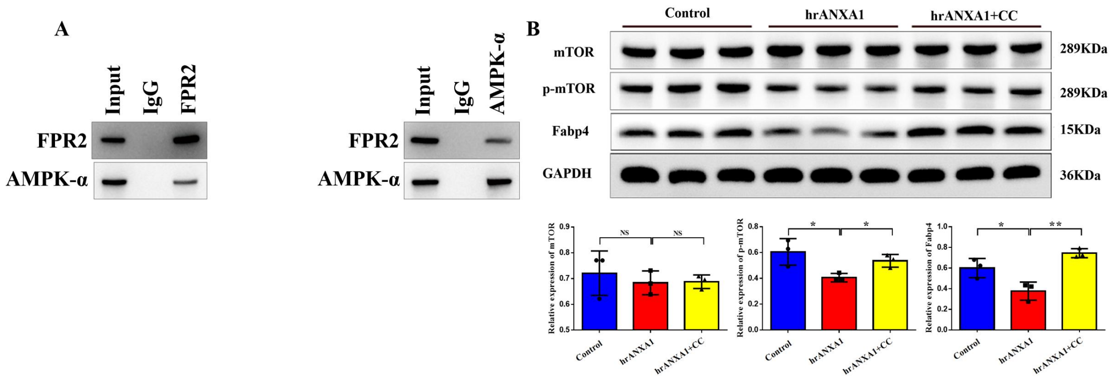

Figure S10. FPR2/ALX regulates mTOR/FABP4 signaling and involves AMPK.

(A) Co-immunoprecipitation assays showing the interaction between FPR2 and AMPK-α. The left panel demonstrates that AMPK-α is co-immunoprecipitated with FPR2 using an anti-FPR2 antibody. The right panel shows that FPR2 is co-immunoprecipitated with AMPK-α using an anti-AMPK-α antibody. IgG serves as a negative control, and Input represents the total protein lysate. (**B)** Representative western blot bands and densitometric quantification of the expression of Fabp4 and the phosphorylation of the mammalian target of rapamycin (mTOR) in BMDMs treated with 10 nM hrANXA1 for 24h with or without the AMPK inhibitor Compound C (CC). n = 3 per group. Data analyses were performed by Student's *t*-test for two groups. *$P < 0.05$; **$P < 0.01$.

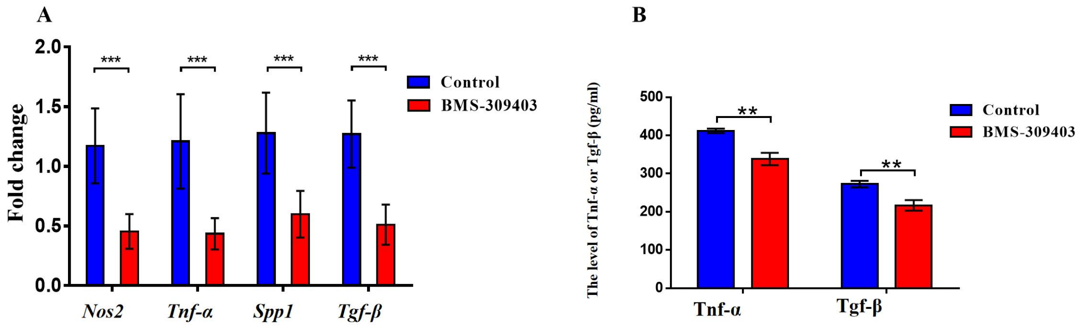

Figure S11. Pharmacological inhibition of Fabp4 in BMDMs from *Anxa1*-deficient mice.

(A) mRNA expression levels of proinflammatory (*Nos2*, *Tnf-α*) and profibrotic (*Spp1*, *Tgf-β*) genes in BMDMs from *Anxa1* knockout mice treated with the Fabp4 inhibitor BMS-309403 (10 μM). n = 9 per group. (**B)** Secreted levels of Tnf-α and Tgf-β in culture supernatants measured by ELISA. n = 3 per group. Data analyses were performed by Student's *t*-test for two groups. $^{**}P < 0.01$; $^{***}P < 0.001$.

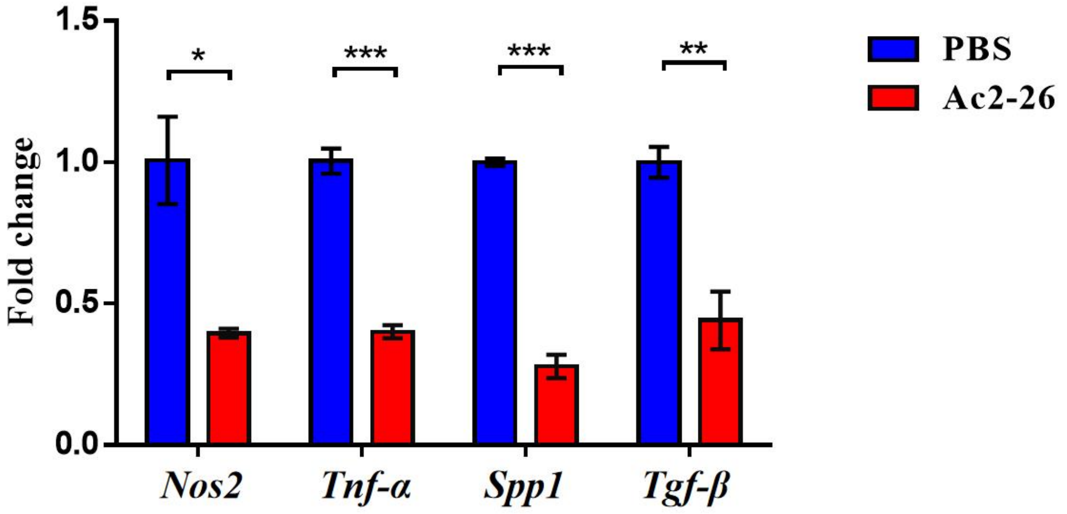

Figure S12. Quantitative real-time polymerase chain reaction (RT-qPCR) analysis of *Nos2*, *Tnf-α*, *Spp1*, and *Tgf-β* mRNA levels in kidney tissues from 20-week-old MRL/*lpr* mice treated with or without Ac2-26. n = 3 per group. Data analyses were performed by Student's *t*-test for two groups. $^{*}P < 0.05$; $^{**}P < 0.01$; $^{***}P < 0.001$.

3. Supplementary Tables

Table S1. Antibodies and materials used in the current study.

Table S2. Clinical characteristics of patients with lupus nephritis at the time of biopsy

At the time of the biopsy	
**Clinical Evaluation**	
Number of patients	33
Age (median and interquartile range) (years)	32 (25-39)
Female (%)	27 (81.8)
**Laboratory Assessment**	
Serum creatinine value (median and interquartile range) (μmol/L)	70.0 (59.5, 123.0)
eGFR (median and interquartile range) (ml/min/1.73m^2)	102.3 (48.8, 129.9)
Urine protein amount (median and interquartile range) (g/24 h)	5.1 (2.3, 7.6)
C3 level (median and interquartile range) (g/L)	0.53 (0.31, 0.72)
C4 level (median and interquartile range) (g/L)	0.11 (0.07, 0.18)
**Renal Histopathology data**	
Histologic classes, n (%)	
I	0 (0.0)
II	1 (3.0)
III(III/III+V)	6 (18.2)
IV(IV/IV+V)	24 (72.7)
V	2 (6.1)
VI	0 (0.0)
IgG deposition, n (%), 1+/2+/3-4+	4 (12.1%)/6 (18.2%)/22 (66.7%)
IgA deposition, n (%), 1+/2+/3-4+	3 (9.1%)/13 (39.4%)/11 (33.3%)
IgM deposition, n (%), 1+/2+/3-4+	14 (42.4%)/15 (45.5%)/0 (0.0%)
C1q deposition, n (%), 1+/2+/3-4+	6 (18.2%)/13 (39.4%)/10 (30.3%)
C3c deposition, n (%), 1+/2+/3-4+	4 (12.1%)/7 (21.2%)/20 (60.6%)

Notes: eGFR: estimated glomerular filtration rate

Table S3. Associations between tubulointerstitial ANXA1 expression and clinicopathological features of patients with lupus nephritis

	tubulointerstitial ANXA1 expression	
**Clinical features**		***P* value**
Hypertension (No/Yes)	0.001 (0, 0.005)/0.002 (0, 0.005)	0.194
NS (No/Yes)	0.004 (0.001, 0.011)/0.002 (0, 0.005)	0.424
AKI (No/Yes)	0.001 (0, 0.003)/0.005 (0.001, 0.016)	0.013
Hematuria	0.001 (0, 0.003)/0.002 (0, 0.007)	0.175
Leukocyturia (noninfectious)	0.002 (0, 0.009)/0 (0, 0.003)	0.112
Anti-SSB antibody (No/Yes)	0.002 (0, 0.005)/0 (0, 0.002)	0.048
	**r value**	***P* value**
Age (years)	0.446	0.009
SLEADI	-0.059	0.743
Hb (g/L)	-0.071	0.695
Proteinuria (g/day)	0.208	0.269
Serum creatinine (μmol/L)	0.258	0.147
C3 level (g/L)	0.080	0.659
C4 level (g/L)	-0.032	0.859
Anti-dsDNA antibodies	-0.149	0.440
Anti-C1q antibodies	-0.471	0.009
**Renal histopathologic features (light microscopy)**		***P* value**
Histologic classes (nonproliferative/proliferative)	0 (0, 0.001)/0.002 (0, 0.005)	0.037
Neutrophils exudation/karyorrhexis (No/Yes)	0.001 (0, 0.002)/0.002 (0, 0.006)	0.129
Fibrinoid necrosis (No/Yes)	0.002 (0, 0.011)/0.001 (0, 0.005)	0.471
Mesangial hypercellularity (No/Yes)	0.001 (0, 0.002)/0.002 (0, 0.011)	0.151
Endocapillary hypercellularity (No/Yes)	0.001 (0, 0.005)/0.002 (0, 0.008)	0.398
Hyaline deposits (No/Yes)	0.003 (0, 0.013)/0.002 (0, 0.004)	0.843
	**r value**	***P* value**
Cellular/fibrocellular crescents	0.419	0.017
Interstitial inflammation	0.353	0.044
CI	0.632	0.002
Glomerulosclerosis	0.688	<0.001
Tubular atrophy	0.499	0.003
Interstitial fibrosis	0.300	0.090
**Renal histopathologic features (direct immunofluorescence)**		***P* value**
IgG deposition (⩽2+/>2+)	0 (0, 0.005)/0.002 (0, 0.005)	0.458
IgA deposition (⩽2+/>2+)	0.002 (0, 0.005)/0.002 (0, 0.007)	0.650
C3c deposition (⩽2+/>2+)	0.002 (0, 0.005)/0.001 (0, 0.005)	0.910
C1q deposition (⩽2+/>2+)	0.002 (0, 0.005)/0.002 (0, 0.003)	0.907

Notes: NS: nephrotic syndrome; AKI: acute kidney injury is defined as any of the following, on the basis of the KDIGO criteria: an increase in serum creatinine of ×0.3 mg/dl (×26.5 μmol/L) within 48 hours or an increase in serum creatinine to ×1.5 times baseline, which is known or presumed to have occurred within the previous 7 days, or urine volume <0.5 ml/kg per hour for 6 hours; SLEDAI: systemic lupus erythematosus disease activity index; Hb: hemoglobin; dsDNA: double-stranded DNA. AI: NIH activity index; CI: NIH chronicity index. Nonparametric variables are expressed as medians (ranges) and were compared *via* the Mann–Whitney test. Correlations were carried out *via* the Spearman test.

Table S4. A total of 37,143 cells were further divided into 14 subgroups on the basis of typical monocyte/macrophage markers.

Table S5. Summary of the proportions of assigned monocyte/macrophage types in the pre-diseased and nephritic groups

Number	Name	Pre-diseased group		Nephritic group	
		Frequency	Ratio	Frequency	Ratio
1	*Slamf9⁺Cxcl2⁻*	5281	27.97%	3900	21.35%
2	*Mmp13⁺Ccl7⁺*	5096	26.99%	3978	21.78%
3	*Cd300e⁺*	2266	12.00%	3650	19.98%
4	*Mki67⁺*	1574	8.34%	742	4.06%
5	*Anxa1⁺Spp1⁺*	322	1.71%	1981	10.85%
6	*Vcan⁺*	908	4.81%	1158	6.34%
7	*Il18 bp⁺*	746	3.95%	1002	5.49%
8	*S100a9⁺*	934	4.95%	367	2.01%
9	*Mmp12⁺*	321	1.70%	598	3.27%
10	*Cd209a⁺*	470	2.49%	148	0.81%
11	*Cfap44⁺*	372	1.97%	200	1.10%
12	*Retnla⁺*	180	0.95%	287	1.57%
13	*Ifit1⁺*	267	1.42%	120	0.66%
14	*Gm10800⁺*	142	0.75%	133	0.73%

Table S6. Summary of the proportions of assigned typical infiltrating and resident macrophage types in the kidney in the pre-diseased and nephritic groups

Number	Name	Pre-diseased group		Nephritic group	
		Frequency	Ratio	Frequency	Ratio
1	*Slamf9⁺Cxcl2⁻*	5160	33.65%	3863	22.73%
2	*Mmp13⁺Ccl7⁺*	5094	33.22%	3977	23.40%
3	*Cd300e⁺*	527	3.44%	3131	18.42%
5	*Anxa1⁺Spp1⁺*	296	1.93%	1961	11.54%
6	*Vcan⁺*	410	2.67%	808	4.75%
7	*Il18 bp⁺*	740	4.83%	1001	5.89%
8	*S100a9⁺*	135	0.88%	180	1.06%
9	*Mmp12⁺*	321	2.09%	598	3.52%

Notes: Not showing the proportion of all 14 macrophage subpopulations.

Table S7. Grading standards for glomerulonephritis.

	Glomeruli
0 (normal)	-
1 (mild)	cell proliferation and/or cell infiltration
2 (moderate)	above + membrane proliferation
3 (severe)	above + crescent formation and/or hyalinosis
